# Probing biotin receptors in cancer cells with rationally designed fluorogenic squaraine dimers†

**DOI:** 10.1039/d0sc01973a

**Published:** 2020-07-09

**Authors:** Kyong T. Fam, Mayeul Collot, Andrey S. Klymchenko

**Affiliations:** Nanochemistry and Bioimaging Group, Laboratoire de Bioimagerie et Pathologies, CNRS UMR 7021, Université de Strasbourg, Faculté de Pharmacie 67401 Illkirch France andrey.klymchenko@unistra.fr mayeul.collot@unistra.fr

## Abstract

Fluorogenic probes enable imaging biomolecular targets with high sensitivity and maximal signal-to-background ratio under non-wash conditions. Here, we focus on the molecular design of biotinylated dimeric squaraines that undergo aggregation-caused quenching in aqueous media through intramolecular H-type dimerization, but turn on their fluorescence in apolar environment due to target-mediated disaggregation. Our structure–property study revealed that depending on the linkers used to connect the squaraine dyes, different aggregation patterns could be obtained (intramolecular dimerization *versus* intermolecular aggregation) leading to different probing efficiencies. Using a relatively short l-lysine linker we developed a bright fluorogenic probe, **Sq2B**, displaying only intramolecular dimerization-caused quenching properties in aqueous media. The latter was successfully applied for imaging biotin receptors, in particular sodium-dependent multivitamin transporter (SMVT), which are overexpressed at the surface of cancer cells. Competitive displacement with SMVT-targets, such as biotin, lipoic acid or sodium pantothenate, showed **Sq2B** targeting ability to SMVT. This fluorogenic probe for biotin receptors could distinguish cancer cells (HeLa and KB) from model non-cancer cell lines (NIH/3T3 and HEK293T). The obtained results provide guidelines for development of new dimerization-based fluorogenic probes and propose bright tools for imaging biotin receptors, which is particularly important for specific detection of cancer cells.

## Abstract 2

Rational design of self-quenched squaraine dimers bearing biotin yielded a bright fluorogenic probe that can distinguish cancerous from non-cancerous cells.

## Graphical abstract

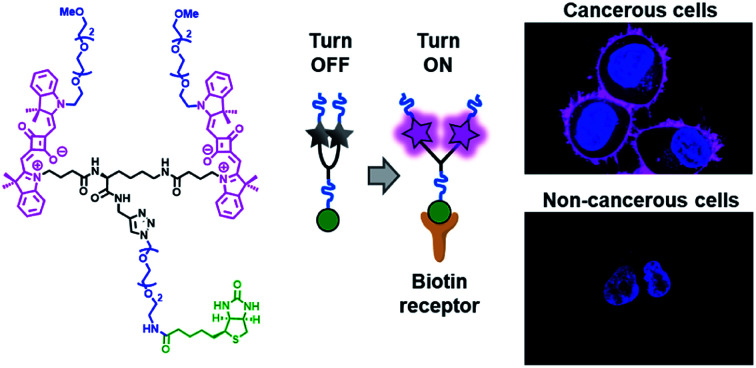

## Introduction

Biotin is an essential vitamin playing its role in cellular carbohydrate, amino acid and lipid metabolism.^1^ Unlike bacteria, mammalian cell machinery does not produce biotin, therefore biotin is supplemented exogenously.^1^ There is evidence that expression of biotin receptors (BRs) is correlated with cancer.^2^ Among BRs, sodium-dependent multivitamin transporter (SMVT) is essential to deliver vitamins, like biotin, to cancer cells.^3^ Therefore, SMVT is a potentially useful cancer biomarker for tumor diagnostics. While BRs have already been exploited for specific targeting of cancer cells,^3,4^ there is lack of robust imaging probes for BRs (in particular SMVT) in cancerous cells and evaluation of new targeted therapeutics.

**Fig. 1:**
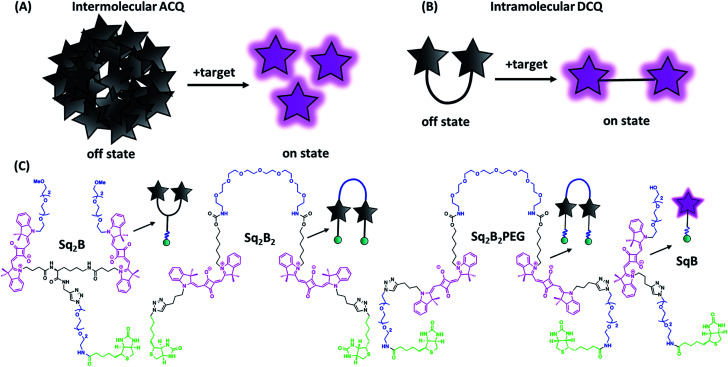
Aggregation-caused quenching (ACQ) in fluorogenic probe design. (A) Schematic representation of ‘turn-on’ fluorescence of a probe based on intermolecular ACQ approach upon binding to the target. (B) Schematic representation of ‘turn-on’ fluorescence of a probe based on intramolecular dimerization-caused quenching (DCQ) approach upon binding to the target. (C) Chemical structures of biotinylated fluorogenic squaraines.

Fluorogenic probes are particularly adapted for deciphering biological processes with background-free imaging.^5–11^ Although environment-sensitive biotin probes have been developed for SMVT imaging operating in the visible region,^3,4,12–14^ detection of SMVT at low concentration requires superior brightness. Alternatively, recent efforts resulted in development of hybrid nanoprobes to distinguish SMVT in cancer cells and 3D spheroids.^15–18^ There is a particular demand in development of probes operating in far-red and near-infrared (NIR) region to image deeper in the tissues and potentially *in vivo*.^19–22^ A number of concepts to generate fluorogenic response to target-binding was developed in the last decade.^6^ First, one should mention push–pull dyes, which are poorly fluorescent in water, but light up in apolar environments of cellular receptors.^23,24^ Second, molecular rotors,^25^ which are poorly fluorescent in aqueous media, but light up upon binding to the targets with highly viscous environment.^26,27^ One should also mention aggregation-induced emission approach, where the non-emissive dyes light up because the target triggers dye aggregation.^28^ Of particular interest are probes operating by aggregation-caused quenching (ACQ) mechanism.^29^ ACQ is caused by strong hydrophobic effects and π–π stacking of dyes at high concentrations.^30^ ACQ is common for fluorophores in aqueous media since their planar hydrophobic aromatic systems provoke formation of non-radiative excimers/exciplexes.^30^ Aggregation is thermodynamically favorable in water, but it could be disrupted in an appropriate environment.^30^ Over the years, a variety of fluorogenic probes has been developed, where the initially quenched aggregated dye species are spatially separated upon interaction with the biological target (Fig. 1A).^29^ Both intermolecular and intramolecular aggregation processes have been used to design ACQ-driven probes. Hamachi and colleagues introduced a ACQ fluorogenic probes with tethered ligand to study protein interaction.^31^ To promote self-aggregation of hydrophilic dyes, they introduced hydrophobic module between fluorophore and a target-recognition unit to obtain turn-on probes for visualization of folate receptor and hypoxia-inducible membrane-bound carbonic anhydrases on the surface of cancer cells.^32^ Later, Tan and colleagues exemplified the utility of target-mediated disaggregation of ACQ cyanine-conjugate to image cell surface proteins.^33^ To obtain fluorogenic probes for plasma membranes, we designed amphiphilic dyes that assembled in water into non-fluorescent ACQ-particles, whereas upon binding to cellular membranes they disassembled activating fluorescence.^34–37^

On the other hand, intramolecular dimerization-caused quenching (DCQ) strategy opens opportunity to control proximity of dyes that can form non-emissive monomolecular self-aggregates in a concentration-independent manner (Fig. 1B). DCQ strategy allows to use lower concentration of probes. Early examples of DCQ probes include YOYO-1,^38^ TOTO-1 and ECHO^39^ probes that fluoresced only upon intercalating in DNA strand. Also, Packard *et al.* developed a series of DCQ probes targeting the serine protease elastase activity.^40–43^ Knemeyer *et al.* developed a hairpin-structured oligonucleotide DCQ probe that increased its fluorescence only upon hybridization to the target DNA.^44^ Recently, we reported a cell-permeant DCQ probe that lighted up in complex with an engineered aptamer, allowing live-cell imaging of intracellular RNA.^45^

Squaraines are particularly interesting for probe development since they have exceptional molar extinction coefficient (∼300 000 M^−1^ cm^−1^) and their absorption and emission are in the far-red to NIR window.^46,47^ In aqueous environment, due to high lipophilic nature squaraines tend to self-aggregate forming non-fluorescent species caused by ACQ phenomenon.^6^ Recently, we combined two squaraine units into one molecule, which folded in aqueous media into an intramolecular self-quenched dimer. Attachment of a receptor-specific ligand to a dimeric squaraines resulted in DCQ fluorogenic probe that turned on its fluorescence on binding specifically to membrane receptor, oxytocin.^48^ Later on, Yao *et al.* reported a NIR DCQ probe based on unsymmetrical squaraines that could selectively respond to cancer biomarker lysophosphatidic acid.^49^

In this work, we rationally designed and synthesized a series of biotinylated fluorogenic squaraines. We took advantage of DCQ strategy to study structure–properties relationship to show its benefits over always-on monomeric probe. We identified a DCQ probe capable to detect cellular BRs (predominantly SMVT) in a bright and specific manner.

## Materials and methods

### Synthesis

The synthesis, protocols, characterizations, and spectra are described in the ESI.† NMR spectra were recorded on a Bruker Avance III 400 MHz spectrometer. Mass spectra were obtained using an Agilent QTOF 6520 mass spectrometer.

### Spectroscopy

Absorption spectra were recorded on a Cary 5000 spectrophotometer (Varian) and fluorescence spectra on a FS5 (Edinburgh Instruments) spectrofluorometer. The fluorescence signal was corrected for the lamp intensity fluctuations and for wavelength-dependent sensitivity of the detector. Relative fluorescence quantum yields were measured using DiD in methanol^50^ (QY = 0.33) as standard.

### Microscopy imaging

Cells were grown at 37 °C in humidified atmosphere containing 5% CO_2_: KB cells (ATCC® CCL-17) in Dulbecco's Modified Eagle Medium without phenol red (DMEM, Gibco-Invitrogen) with 10% fetal bovine serum (FBS, Lonza), 1% non-essential amino acids (Gibco-Invitrogen), 1% MEM vitamin solution (Gibco-Invitrogen), 1% l-glutamine (Sigma Aldrich) and 0.1% antibiotic solution (gentamicin, Sigma-Aldrich); HEK293T (ATCC® CRL-3216™) and HeLa (ATCC® CCL-2™) in DMEM without phenol red supplemented with 10% FBS (Lonza), 1% l-glutamine (Sigma Aldrich) and 1% antibiotic solution (Penicillin–Streptomycin, Sigma-Aldrich); NIH/3T3 (ATCC® CRL-1658™) in DMEM without phenol red supplemented with 10% bovine calf serum, iron fortified (Sigma Aldrich), 1% l-glutamine (Sigma Aldrich) and 1% antibiotic solution (Penicillin–Streptomycin, Sigma-Aldrich). Cells were seeded onto a chambered coverglass (IBiDi®) 24 h before the microscopy measurement. For imaging, the culture medium was removed, the attached cells were washed with Hank's Balanced Salt Solution (HBSS, Gibco-Invitrogen) and incubated with solution of **Sq2B** (0.2 μM). In competition experiment, KB cells were pretreated with competitor (100 μM) for 30 min prior to incubation with **Sq2B** probe. Images were taken with Nikon Ti-E inverted microscope, equipped with CFI Plan Apo × 60 oil (NA = 1.4) objective, X-light spinning disk module (CresOptics) and a Hamamatsu Orca Flash 4 sCMOS camera, was used. The microscopy settings were: Hoechst (ex. 405 nm, em. 510 ± 42 nm), squaraine (ex. 638 nm, em. 705 ± 36 nm). The images were recorded using NIS elements and then processed with Icy open source imaging software.

### Flow cytometry

Cells were grown at 37 °C in humidified atmosphere containing 5% CO_2_ in 25 cm^2^ (Nunc™ EasYFlask™, ThermoFisher). On the day of the analysis, the cells were washed and harvested. The cell suspension (3 × 10^5^ cells per mL) was incubated with corresponding **Sq2B** probe (0.2 μM) for 5 min at room temperature and analyzed immediately using flow cytometry (MACSQuant VYB, Miltenyi Biotec).

### Cytotoxicity assay

Cytotoxicity of the dyes was quantified by the MTT assay (3-(4,5-dimethylthiazol-2-yl)-2,5-diphenyltetrazolium bromide). A total of 1 × 10^4^ KB cells per well were seeded in a 96-well plate 24 h prior to the cytotoxicity assay in growth medium and were incubated in a 5% CO_2_ incubator at 37 °C. After medium removal, an amount of 100 μL DMEM containing 5 μM, 1 μM or 0.2 μM of a probe was added to the KB cell and incubated for 24 h at 37 °C (5% CO_2_). As control, for each 96-well plate, the cells were incubated with DMEM containing the same percentage of DMSO (0.5% v/v) as the solution with the tested dyes. After 24 h of dye incubation, the medium was replaced by 100 μL of a mix containing DMEM + MTT solution (diluted in PBS beforehand) and the cells were incubated for 4 h at 37 °C. Then, 75 μL of the mix was replaced by 50 μL of DMSO (100%) and gently shaken for 15 min at room temperature in order to dissolve the insoluble purple formazan reduced in living cells. The absorbance at 540 nm was measured (absorbance of the dyes at 540 nm were taken into account). Data were shown as the mean value (*n* = 6) plus a standard deviation (±SD). For each concentration, we calculated the percentage of cell viability in reference of the control DMEM+0.5% DMSO.

## Results and discussion

### Synthesis of biotinylated squaraine dimers

In order to find the optimal design of fluorogenic dimer, we considered two types of dimeric probes. The first one is Y-structure, where two squaraine (Sq.) unites are connected to lysine linker, which was further coupled to a biotin ligand (**Sq2B**, Fig. 1C). In contrast to our originally proposed system for GPCR receptor,^48^ the present design is simpler with shorter distance between the Sq dyes and it bears small biotin ligand instead of cyclic peptide. A monomeric dye analogue of this probe (**SqB**, Fig. 1C) has also been obtained as a control. In the second design strategy, we developed symmetrical U-shape dimers of squaraines connected with a flexible PEG8-bridge and bearing two biotin moieties (**Sq2B2**, **Sq2B2PEG**, Fig. 1C). Activated carbonate **5** (Schemes S1 and S2 ESI†) was coupled to PEG8-diamine resulting in **6** with two propargyl handles (Scheme S2, ESI†), which were further clicked to N_3_-biotin or N_3_-PEG3-biotin, respectively (Scheme S3, ESI†). We assumed that introduction of additional short PEG-linker between biotin and Sq unit in **Sq2B2PEG** would increase the solubility of the probe in aqueous solutions as well as add the degree of flexibility to dynamically fold and prevent nonspecific binding to biomolecules.

### Fluorogenic properties of squaraine dimers

**Fig. 2:**
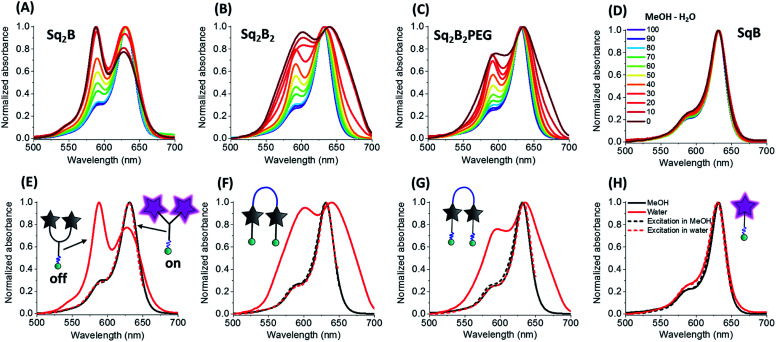
Photo-physical properties of biotinylated squaraine probes. Normalized absorption spectra of **Sq2B** (A), **Sq2B2** (B), **Sq2B2PEG** (C) and **SqB** (D) in the gradient mixture of MeOH–H_2_O (from 100/0 to 0/100). Normalized absorption and excitation spectra in MeOH and water of **Sq2B** (E), **Sq2B2** (F), **Sq2B2PEG** (G) and **SqB** (H). Concentration of probes was fixed to 0.2 μM.

To evaluate the fluorogenic nature of the dimers, we studied their photophysical properties at a fixed concentration (0.2 μM) in the mixture of water with gradual increase of organic solvent content (MeOH). In MeOH, all four probes exhibited absorption spectra typical for monomeric fluorophore (Fig. 2), characterized by narrow bands with maxima at ∼630 and ∼640 nm, respectively.

With increase of the water fraction in methanol, the monomeric squaraine **SqB** did not show any significant changes in the absorption spectra, indicating that at 0.2 μM it did not aggregate (Fig. 2D). The absence of aggregation of monomeric **SqB** can be attributed to high degree of PEGylation that ensures its water solubility. On the other hand, all dimers showed formation of blue-shifted band at higher water fractions (Fig. 2A–C). This new band was absent in the fluorescence excitation spectra of all dimers (Fig. 2E–G) the latter being superposed with absorption spectra in methanol, indicating that this new blue shifted absorption band was non-emissive. According to our previous studies,^48^ this new band can be assigned to intramolecular H-aggregates of squaraines.

However, at the highest water fractions and in neat water, the shape of the absorption spectra of dimers depended on their chemical structure (Fig. 2A–C). Indeed, in neat water Y-dimer **Sq2B** showed typical sharp blue shifted H-aggregate absorption band centred at 588 nm and a peak at 632 nm (Fig. 2E), whereas for U-dimers **Sq2B2** and **Sq2B2PEG** displayed broad absorption spectra (Fig. 2F and G). We assumed that the difference for U-probes originated from their aggregation behaviour: intra-, intermolecular aggregation or the synergy of both phenomena.

**Fig. 3:**
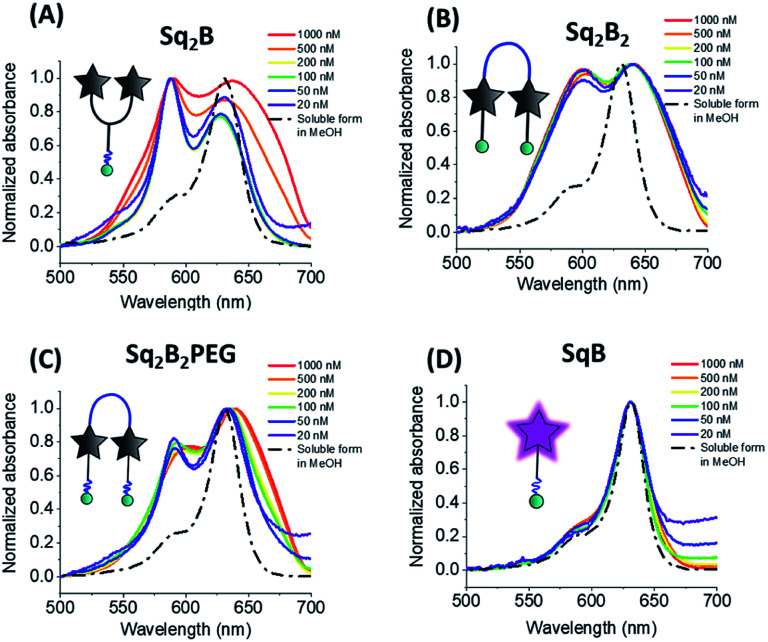
Absorption spectra of **Sq2B** (A), **Sq2B2** (B), **Sq2B2PEG** (C) and **SqB** (D) at different concentrations in water. For comparison normalized absorbance spectra of probes in soluble form in methanol are shown in black dashed line.

To decipher the nature of the aggregates, we studied the absorption spectra of the squaraines at different concentrations (20 nM to 1 μM) in water and compared to the spectra of their solutions in MeOH (Fig. 3). Lysine-based Y-dimer **Sq2B** displayed sharp absorption bands that did not change their relative intensity up to 200 nM (Fig. 3A). This result confirmed the intramolecular nature of the formed H-aggregate, which should not be concentration dependent. However, at higher concentrations, the bands of **Sq2B** broadened, indicating a formation of more complex (intermolecular) aggregates. In case of U-dimer **Sq2B2PEG** at low concentration, the bands were also relatively sharp and concentration-independent, although the H-aggregate band were less intense (Fig. 3C). Thus, similar to **Sq2B**, **Sq2B2PEG** can also form intramolecular H-aggregate, but its fraction was lower probably because its squaraine units have higher degree of freedom in case of PEG8-based linker as compared to lysine linker of **Sq2B**. At concentrations above 100 nM **Sq2B2PEG** showed band broadening probably due to intermolecular aggregation. By contrast, U-dimer without PEG3 units, **Sq2B2**, displayed broad absorption spectra at all studied concentrations (Fig. 3B), which is probably caused by lipophilic nature of the molecule that favours the intermolecular aggregation even at low concentration. Unlike dimeric variants, monomeric **SqB** showed similar absorption spectrum in water independent of concentration close to that in methanol and, thus preserving non-aggregated form throughout the studied concentration range (Fig. 3D). The fluorescence intensity of monomeric **SqB** increased linearly with dye concentration in water, whereas other dyes showed non-linear saturation behaviour (Fig. S1†), confirming that at high concentration they form intermolecular aggregates.

**Fig. 4:**
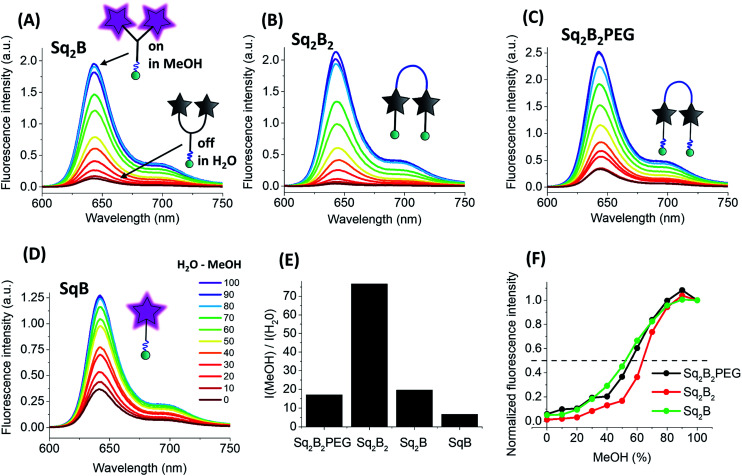
Photo-physical properties of biotinylated squaraine probes. Fluorescence spectra of **Sq2B** (A), **Sq2B2** (B), **Sq2B2PEG** (C) and **SqB** (D) in the gradient mixture of MeOH–H_2_O (from 100/0 to 100/0). (E) Fluorescence enhancement from water to MeOH of **Sq2B**, **Sq2B2**, **Sq2B2PEG** and **SqB**. (F) Fluorescence intensity of the dimers in MeOH/H_2_O mixtures at different fractions of MeOH, normalized to that in neat MeOH. Concentration of probes was fixed to 0.2 μM.

All three dimers showed good fluorescence quantum yield (QY) in methanol (0.15–0.25), whereas in water they were poorly emissive (QY = 0.001–0.01, Table 1). With increase in the fraction of methanol in water the fluorescence intensity of all dimers increased drastically without changing their maximum (Fig. 4A–C). This solvent-dependent fluorogenic behaviour of the dimers clearly resulted from the non-emissive H-aggregates in water, unfolding and turning on their emission in methanol. By contrast, monomer **SqB** showed a non-negligible QY in water (Table 1), so that the increase in its fluorescence with increase of the methanol fraction was less pronounced (Fig. 4D) and was probably linked to an intrinsic fluorogenic property of squaraine.^51,52^ Overall, the dye dimers showed >15-fold fluorescence increase from water to methanol with the strongest change for **Sq2B2**, being much higher than that for monomeric **SqB** (Fig. 4E). The normalized titration curves revealed that the increase of intensity for all dimers was sigmoidal, reflecting a complex non-linear process combining dimer dissociation and direct effect of the solvent on the dye. Nevertheless, **Sq2B** exhibited fluorescence enhancement for lower methanol fractions (curve shifted to the left, Fig. 4F). This difference in the sensitivity to methanol correlates with different aggregation behaviour of the dyes. Indeed, at the low methanol content (*i.e.* high water fractions with 0.2 μM dye) only **Sq2B** is present in form of monomolecular H-aggregate (Fig. 2A). The latter was probably more sensitive to low fractions of methanol that opened this monomolecular dimer, compared to the other two dyes that formed in these conditions intermolecular aggregates (Fig. 2B and C). In addition, we cannot exclude some differences in the preferential solvation of the dye dimers, which can additionally favour opening of **Sq2B** dimer at lower methanol concentration. Taken together, only **Sq2B** operated by intramolecular DCQ mechanism while fluorogenic properties of **Sq2B2** and **Sq2B2PEG** were characterized by more complex aggregation, synergic intramolecular DCQ and intermolecular ACQ.

**Table 1:** Photo-physical properties of biotinylated squaraine probes

Dye	Solvent	*Φ* a	Brightness^b^	Relative brightness^c^	*Φ*_enh._
**Sq2B**	MeOH	0.15	99 000	2.31	50
	H_2_O	0.003	—	—	
**Sq2B2PEG**	MeOH	0.25	165 000	3.85	25
	H_2_O	0.01	—	—	
**Sq2B2**	MeOH	0.18	118 800	2.77	180
	H_2_O	0.001	—	—	
**SqB**	MeOH	0.13	42 900	1	3.82
	H_2_O	0.034	—	—	

a Quantum yields (QY) were measured using reference DiD dye in methanol (*Φ* = 0.33).^50^

b Brightness was calculated as *ε* × *Φ*, *ε* = 330 000 M^−1^ cm^−1^ for monomer in methanol^48^ and *ε* = 660 000 M^−1^ cm^−1^ for dimers.

c Relative brightness to **SqB** in methanol. *Φ*_enh._ is an enhancement in the quantum yield with respect to water.

In the next step, we evaluated potential nonspecific interactions of the biotin conjugates with biological components: bovine serum albumin (BSA), fetal bovine serum (FBS), biotin-binding proteins (streptavidin and avidin), biotin and lipid vesicles composed of DOPC. To this end, we measured fluorescence intensity of the probes, which was expected to increase in case of nonspecific interaction with these components, and compared it to the signal of activated probes in methanol (Fig. S2†). To our surprise, each probe responded differently to these model systems (Table 2). Probes **Sq2B** and **Sq2B2PEG** showed lower degree of nonspecific fluorescence turn-on response in comparison to more lipophilic **Sq2B2**.

**Table 2:** Non-specific fluorescence turn-on by interaction with biomolecules

% of max F.I.^a^	**Sq2B2PEG**	**Sq2B2**	**Sq2B**	**SqB**
MeOH	100	100	100	100
H_2_O	13.30	2.067	7.47	28.93
PBS	6.96	2.060	2.098	16.99
BSA	40.075	31.17	38.56	243.50
FBS	18.36	8.20	9.084	127.91
Avidin	6.83	2.31	1.98	21.18
Streptavidin	7.075	2.56	1.51	11.75
Biotin	7.25	2.24	4.56	19.74
DOPC vesicles	21.33	7.81	17.32	91.35

a F.I. is fluorescence increase. Concentration of probes was fixed to 0.2 μM. Concentrations of biomolecules: BSA (0.1 mg mL^−1^), FBS (0.1 mg mL^−1^), avidin (100 nM), streptavidin (100 nM), biotin (100 μM) or DOPC vesicles (20 μM).

Generally, all dimers showed strong fluorescence increase in the presence of BSA (31–40% of open form, Table 2 and Fig. S2†). Surprisingly, **SqB** was more than twice more fluorescent in the presence of BSA in comparison to its emission in MeOH. Such a behaviour is probably caused by binding of the dye into the hydrophobic pocket of the albumin, where the intramolecular rotation of the molecules is restricted, favouring strong emission of the dye. Importantly, in FBS, which is the biological medium rich in proteins and lipoproteins, **Sq2B2PEG**, **Sq2B2** and **Sq2B** showed low fluorescence activation in comparison to signal in PBS, but FBS significantly increased emission of **SqB**. Unexpectedly, interaction with biotin-binding proteins (avidin and streptavidin) did not increase fluorescence of all four probes. Having two biotin units, **Sq2B2** and **Sq2B2PEG** were expected to bind two neighbouring binding sites of avidin/streptavidin to promote disassembly of Sq units and, thus, increase their fluorescence. Judging by our data, we assume that **Sq2B2** and **Sq2B2PEG** bound only one site of the proteins with a single biotin unit while favourable DCQ phenomenon kept dimers in closed form. As expected, the presence of free biotin did not contribute to unspecific fluorescence. In case of DOPC vesicles, the general trend was the same as for FBS, suggesting low interaction of dimers with DOPC or absorption on the surface in a closed form. Unlike dimers, fluorescence of **SqB** increased in the presence of DOPC reaching the value close the one in methanol, probably due to restriction of molecular rotation **SqB** in the lipid bilayer. Overall, these data suggest that, unlike always-on **SqB** probe, DCQ biotin probes had reduced untargeted interaction with biological components.

### Imaging of biotin receptors with **Sq2B**

**Fig. 5:**
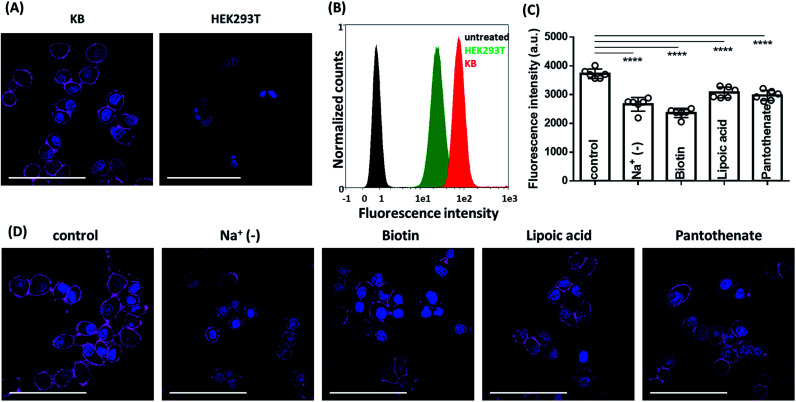
Fluorescence microscopy images. (A) Live cell fluorescence imaging of **Sq2B** (0.2 μM) in BR-positive (KB) and BR-negative (HEK293T) cells. (B) Flow cytometry analysis of KB and HEK293T cell treated with **Sq2B**. Untreated KB cells were used as negative control. (C) Analysis of fluorescence intensities from images (D). Bars represent mean value ± SD (*n* = 6). Statistical significance based on unpaired *t*-test analysis: ****p < 0.0001. (D) Fluorescence imaging of **Sq2B** in KB cells in Na^+^-deprived HBSS, pre-treated with biotin (100 μM), lipoic acid (100 μM) or pantothenate (100 μM) compared to control. In all cases, the probe was incubated for 5 min at rt. Nuclei were labelled with Hoechst (5 μg mL^−1^). Scale bar, 100 μm.

After characterisation of spectral properties, we pursued cellular studies. We chose **Sq2B** probe since it showed low unspecific fluorescence with biomolecules as well as retained its water soluble self-quenched monomolecular form in range 20 to 200 nM. Cancer cells, like KB (HeLa cells derivatives) tend to consume biotin in a high extend as vital supplement in comparison to other cells suggesting over expression of BRs.^3^ Therefore, we used KB cells as cellular model to evaluate performance of **Sq2B** and HEK293T cells were used as BR-negative cell line.^3^ The biotinylated **Sq2B** showed low toxicity in KB cells in the range of imaging concentrations (Fig. S3†). Freshly prepared solution in HBSS (0.2 μM) of **Sq2B** was briefly incubated with cells for 5 min at room temperature and imaged without washing. In KB cells, **Sq2B** readily produced distinctive fluorescence labelling of the cell surface, whereas a very weak signal was observed in case of HEK293T cells (Fig. 5A). We additionally evaluated specificity of **Sq2B** to KB cells by flow cytometry. The population of **Sq2B**-treated KB cells exhibited significantly higher brightness compared to HEK293T in the cell-by-cell analysis (Fig. 5B). This result confirmed our observations by microscopy, suggesting that **Sq2B** distinguished KB cells from HEK293T probably because the former is BR-positive. Additionally, microscopy experiment in other cell lines, in particular cancer HeLa and non-cancer NIH/3T3, showed appearance of **Sq2B** fluorescence only in cancer cell lines (Fig. S4†).

Next, we evaluated the specificity of **Sq2B** to BRs in KB cells. SMVT is well characterized BR in cancer cells to deliver biotin.^3^ Except for biotin transport, SMVT is also known to be vital for translocation of essential cofactors like lipoic acid and panthothenic acid.^53^ To evaluate the targeting ability of **Sq2B** to SMVT, we performed a set of competition experiments (Fig. 5D). Firstly, to evaluate dependence of the staining in the presence of extracellular Na^+^ ions, we incubated KB cells with **Sq2B** in Na^+^-deprived HBSS buffer (Na^+^ was replaced with choline). The results showed the decrease in **Sq2B** fluorescence when Na^+^-deprived media was used indicating a strong effect of extracellular sodium ions on the **Sq2B** cellular binding (Fig. 5D). Secondly, KB cells were pre-treated with competitors (biotin, lipoic acid or sodium pantothenate) to saturate SMVT and thus reduce its availability for **Sq2B**. Further brief incubation with **Sq2B** showed the decrease in the fluorescence in competitor-treated KB cells in comparison to untreated control cells (Fig. 5D). These observations suggest that a significant part of the **Sq2B** signal resulted from its binding to SMVT.

**Fig. 6:**
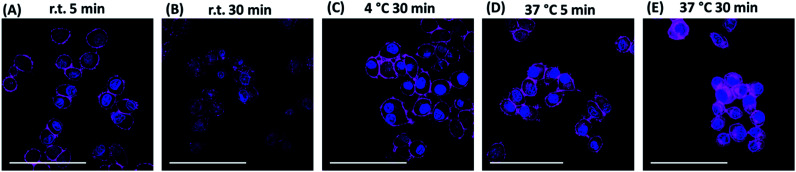
Fluorescence microscopy images of KB cells treated with **Sq2B** (0.2 μM) at different conditions. (A) Incubation for 5 min at room temperature (rt). (B) Incubation for 30 min at rt. (C) Incubation for 30 min at 4 °C. (D) Incubation for 5 min at 37 °C. (E) Incubation for 30 min at 37 °C. Nuclei were labelled with Hoechst (5 μg mL^−1^). Scale bar, 100 μm.

We further studied the internalisation of the probe. When cells were incubated with **Sq2B** at room temperature for 30 min, fluorescence was observed in the cytoplasm (Fig. 6B). Incubation at 37 °C increased the fluorescence inside the cells, indicating faster dynamic interaction with SMVT and higher uptake rate (Fig. 6D and E). However, when KB cells were incubated with probes at 4 °C for 30 min (Fig. 6C) only the cell surface pattern was detected, suggesting the occurrence of binding event only. Absence of the intracellular signal suggested that the dye does not internalize passively into the cells, while the active transport is inhibited by the low temperature. Additionally, Na^+^-depletion in the buffer at 37 °C passivated internalisation of **Sq2B** over 30 min incubation (Fig. S5†). This set of experiments demonstrated that biotin probe **Sq2B** could target overexpressed SMVT on the surface of cancer cells and the internalization of the molecules was caused predominantly by active transport.

Additionally, we compared performance of **Sq2B** with its monomer analogue **SqB** in cell imaging experiments. While **SqB** could stain SMVT in KB cells like **Sq2B**, it internalized stronger in both KB and HEK293T cells. Therefore, the monomeric probe **SqB** produced non-specific signal in BR negative HEK293T cells in comparison to **Sq2B** (Fig. S6A†). Comparative analysis of fluorescence intensity profiles across imaged cells (Fig. S6B†) revealed some additional details. (i) Intense signal at membrane sites was generally 50% higher with **Sq2B***vs.***SqB**, indicating that the dimer probe is brighter. (ii) It was confirmed that, unlike **Sq2B**, **SqB** produced significant intracellular fluorescence in both KB (BR positive) and HEK293T (BR negative) cells. Probably due to its smaller size, monomer **SqB** internalized faster into the cells than the dimer, which made it less selective for BR detection. (iii) **Sq2B** displayed 1.4-fold lower level of background fluorescence compared to that of **SqB**. In line with our spectroscopy data, this experiment confirmed advantage of DCQ probe (**Sq2B**) over non-fluorogenic probe (**SqB**) in terms of brightness, specificity and imaging contrast.

## Conclusions

In conclusion, we designed and synthesized a series of biotinylated fluorogenic probes for biotin receptors overexpressed in cancer cells. We focused on the molecular design of dimeric squaraines operating by ACQ mechanism that enabled tuning the fluorogenic properties while minimizing nonspecific interaction with biomolecules and reducing the background fluorescence.

We made a systematic analysis of structure effect on fluorogenic properties of dimeric squaraines. When designing dimeric fluorogenic dyes following factors should be taken into consideration: (i) close proximity of fluorophores to each other to form self-aggregated monomolecular species in aqueous environment so that the designed probe operates by DCQ mechanism; (ii) hydrophilic linkers to prevent nonspecific binding; (iii) effect of the ligand on inter-/intramolecular interactions. Our structure–properties study revealed that a relatively short l-lysine linker served as a better connector in DCQ-probe design in comparison to PEG8-linker. Additional small PEG-linkers increased water solubility, promoted retention of the monomolecular form while preventing nonspecific interactions in cellular environment.

This strategy enabled us to develop a monomolecular DCQ probe, **Sq2B**, as a promising candidate to report biotin receptor in microscopy and flow cytometry analysis. We showed that **Sq2B** underwent active translocation into the cancer cells at least partially *via* SMVT. In particular, **Sq2B** transport was sodium-dependent and inhibited by native vitamins like biotin, lipoic acid and pantothenic acid.

We believe that this study will provide useful guidelines in designing fluorogenic DCQ probes for bioimaging. The developed probes can be of potential interest for specific detection of cancer cells overexpressing BRs. Additionally, specific **Sq2B** probe will contribute in deciphering the biological role of BRs, in particular SMVT, and built new strategies for evaluation of new therapeutics.

## Conflicts of interest

There are no conflicts of interest to declare.

## Supplementary Material

SC-011-D0SC01973A-s001
