# Supplementary material for: Probing biotin receptors in cancer cells with rationally designed fluorogenic squaraine dimers
Source: Chem Sci. 2020 Jul 9;11(31):8240–8. doi: 10.1039/d0sc01973a (PMC8163205; doi:10.1039/d0sc01973a)
Supplement: SC-011-D0SC01973A-s001 [file SC-011-D0SC01973A-s001.pdf]

Electronic Supplementary Material (ESI) for Chemical Science.
This journal is © The Royal Society of Chemistry 2020

Probing biotin receptors in cancer cells with rationally designed fluorogenic squaraine dimers

Kyong T. Fam,[a] Mayeul Collot*[a] and Andrey Klymchenko*[a]

[a] Nanochemistry and Bioimaging group, Laboratoire de Bioimagerie et Pathologies, CNRS UMR 7021, Université de Strasbourg, Faculté de Pharmacie, 67401 Illkirch, France.

*Corresponding authors: andrey.klymchenko@unistra.fr; mayeul.collot@unistra.fr

Supporting information

PEG7-diamine was synthesized as described elsewhere [1].
N_3-biotin was synthesized according to a published protocol.[2]

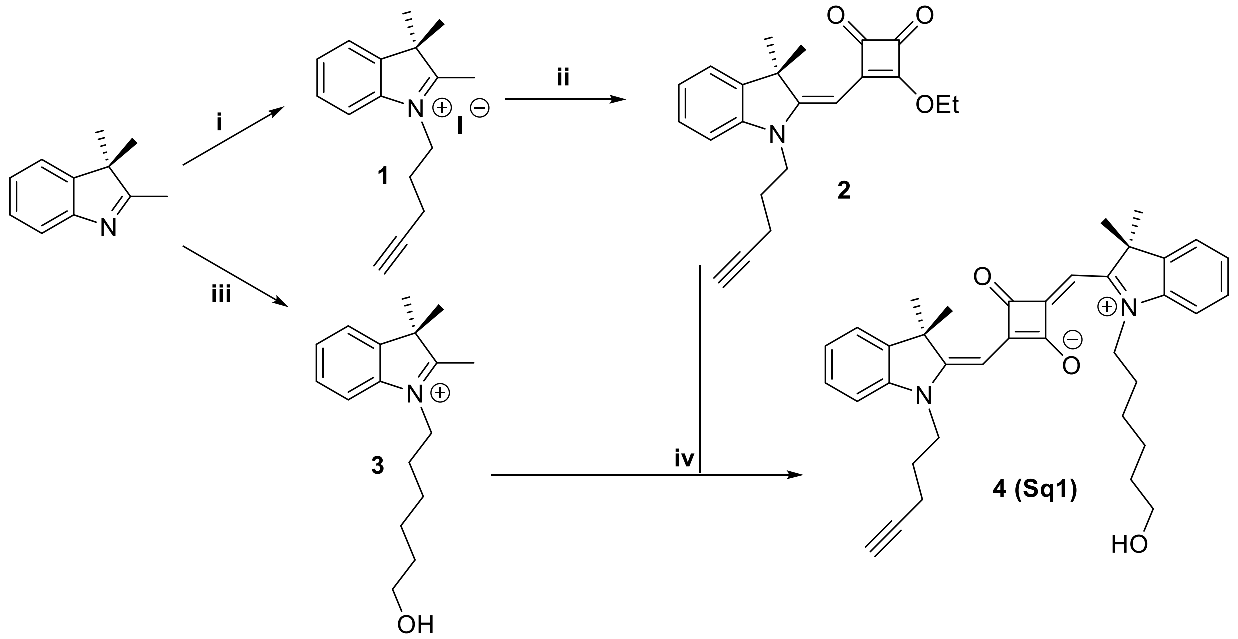

Scheme S1. Synthesis of Sq1. i) 5-chloro-1-pentyne, KI, CH_3CN, 85°C, 48h (20%); ii) Diethyl squarate, Et_3N, EtOH, 80°C, 6h (36%); iii) 6-chloro-1-hexanol, KI, CH_3CN, 110°C, 12h (12%); (iv) pyridine, 125°C, 18h (31%)

Compound **1** was synthesized according to a published protocol.[3]

Synthesis of compound 2. Compound 1 (1.2 g, 3.4 mmol, 1 eq), diethyl squarate (578 mg, 3.4 mmol, 1 eq) and Et_3N (g, mmol, 2.7 eq) were dissolved in EtOH (20 mL). The mixture was refluxed overnight. The solvents were then removed under vacuum and the crude product was purified by column chromatography with Heptane/EtOAc (7/3) as eluent. Yellow solid was obtained, yield 36%.
^{1}H NMR (400 MHz, Chloroform-d) δ 7.25 – 7.16 (m, 2H), 7.01 (td, J = 7.4, 0.9 Hz, 1H), 6.93 – 6.87 (m, 1H), 5.41 (s, 1H), 4.82 (q, J = 7.1 Hz, 2H), 3.91 (t, J = 7.4 Hz, 2H), 2.26 (td, J = 6.7, 2.6 Hz, 2H), 2.06 (t, J = 2.6 Hz, 1H), 1.91 (p, J = 6.9 Hz, 2H), 1.56 (s, 6H), 1.46 (t, J = 7.1 Hz, 3H). ^{13}C NMR (101 MHz, Chloroform-d) δ 192.35, 187.81, 187.72, 173.87, 168.23, 142.60, 140.83, 127.82, 122.75, 122.02, 108.32, 82.69, 81.57, 70.09, 69.93, 47.94, 41.54, 27.06, 25.11, 16.13, 15.92. HRMS (ES+) Calc. for $C_{22}H_{23}NO_3$ $[M+H]^+$ 350.1678, found 350.1757.

Synthesis of compound 3. 2,3,3-trimethylindolenine (1 eq, 9.54g), 6-chloro-1-hexanol (1 eq, 8.16 g) and potassium iodide (2.2 eq, 21.9 g) were dissolved in CH_3CN (75mL), the reaction was heated up to 110°C under stirring overnight. The solvent was then removed under vacuum and the crude product was dissolved in a small amount of acetone. The mixture is then precipitated in cold ether, three times to obtain the maximum of product. Pink crystals were obtained, yield 12%.

^{1}H NMR (400 MHz, Chloroform-d) δ 7.74 – 7.64 (m, 1H), 7.59 – 7.46 (m, 4H), 5.24 (s, 1H), 3.54 (t, J = 5.9 Hz, 2H), 3.07 (s, 3H), 1.93 (t, J = 7.6 Hz, 2H), 1.59 (s, 6H), 1.55-1.42 (m, 8H). ^{13}C NMR (101 MHz, Chloroform-d) δ 195.69, 141.65, 140.94, 130.16, 129.65, 123.35, 115.62, 61.71, 49.88, 32.00, 28.01, 26.31, 25.26, 23.44, 23.26, 17.13.

Synthesis of 4 (Sq1). Compound **3** (244 mg, 0.63 mmol, 1.1 eq) and compound **2** (200 mg, 0.573 mmol, 1 eq) were dissolved in 5 mL of pyridine. After 5 h the solution turns green. The solvent was removed under vacuum and the crude product was purified by column chromatography with EtOAc/MeOH (95/5) as eluent. Blue-green powder was obtained, yield 31%.

^{1}H NMR (400 MHz, Chloroform-d) δ 7.28 (d, J = 1.2 Hz, 1H), 7.26 (d, J = 1.1 Hz, 2H), 7.24 (dd, J = 2.5, 1.2 Hz, 2H), 7.22 (d, J = 1.3 Hz, 2H), 7.20 (d, J = 1.2 Hz, 1H), 7.09 – 7.00 (m, 5H), 6.93 (s, 1H), 6.91 (s, 1H), 5.92 (s, 2H), 3.93 (s, 4H), 3.61 (t, J = 6.1 Hz, 4H), 2.26 (d, J = 2.7 Hz, 2H), 1.95 (s, 3H), 1.79 – 1.72 (m, 3H), 1.69 (d, J = 3.9 Hz, 27H), 1.58 – 1.49 (m, 3H), 1.45 (dh, J = 9.0, 2.3, 1.8 Hz, 6H). ^{13}C NMR (126 MHz, Chloroform-d) δ 179.62, 170.54, 169.73, 142.47, 142.26, 127.85, 127.83, 123.91, 123.71, 122.32, 122.29, 109.49, 109.31, 86.78, 82.71, 70.21, 62.40, 49.37, 49.21, 43.21, 42.23, 32.43, 27.14, 26.98, 26.25, 26.24, 25.62, 24.93, 16.11. HRMS (ES+) Calc. for $C_{37}H_{42}N_2O_3$ $[M+H]^+$ 563.3195, found 563.3265.

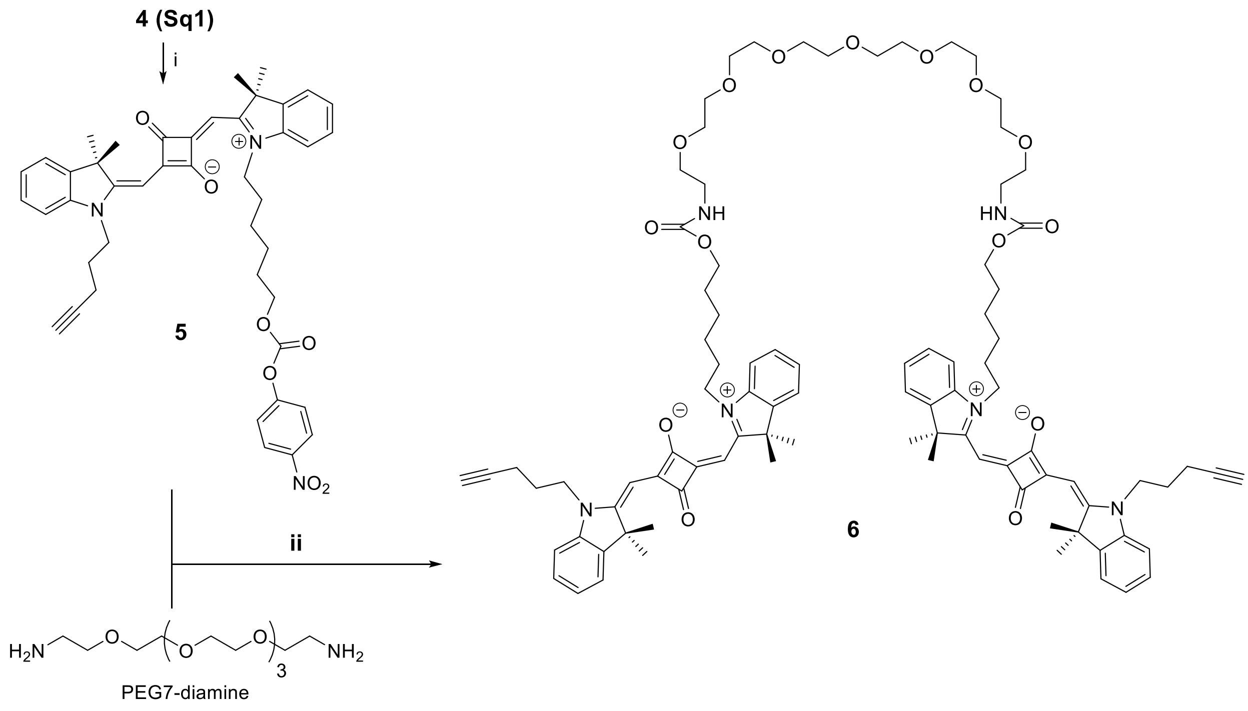

Scheme S2. Synthesis of 6. i) pyridine, DCM, r.t., 3 h (61%); ii) DIEA, DMF, 60°C, 18h (21%);

Synthesis of compound 5. Compound **4** (260 mg, 0.462 mmol, 1 eq) was dissolved in DCM, followed by pyridine (112 µL, 1.386 mmo, 3 eq) addition and 4-nitrophenyl chloroformate (186 mg, 0.924 mmol, 2 eq).

Reaction was left stirring for 3 hours at room temperature. The solvent was evaporated and the residue was purified on column EtOAc/DCM (3/7). Yield, 61%.
^{1}H NMR (400 MHz, Chloroform-d) δ 8.21 (d, 2H), 7.32-7.26 (m, 6H), 7.1-7 (m, 3H), 6.9 (d, 2H), 5.89 (s, 2H), 4.22 (t, 2H), 4.07-4.04 (m, 4H), 2.3 (m, 2H), 2.09 (s, 1H), 1.99 (m, 2H), 1.8 (m, 4H), 1.77 (s, 12H), 1.55 (m, 4H), 1.44 (m, 4H), (d, J = 3.9 Hz, 27H), 1.58 – 1.49 (m, 3H), 1.45 (dh, J = 9.0, 2.3, 1.8 Hz, 6H). ^{13}C NMR (100 MHz, Chloroform-d) δ 127.88, 125.29, 123.82, 109.31 86.65, 69.28, 49.42, 28.43, 27.18, 27.11, 26.99, 25.67, 25.57, 16.17. HRMS (ES+) Calc. for $C_{44}H_{45}N_3O_7$ $[M+H]^+$ 728.3258, found 728.3327.

Synthesis of 6. Compound **5** (60 mg, 2eq) and PEG7-diamine (15 mg, 1 eq) were dissolved in DMF and DIEA (26 mg, 5 eq) was added. The mixture was left to stir overnight at 60°C. The solvent was removed under vacuum and the crude was dissolved in DCM. The solution was washed with concentrated solution of NaNHCO3, dried over MgSO4 and concentrated. The residue was purified by column chromatography with DCM/MeOH (97/3) as eluent. Yield 21%.
^{1}H NMR (500 MHz, Chloroform-d) δ 7.29-7.27 (m, 8H), 7.09-7.02 (m, 6H), 6.91 (d, 2H), 5.88 (s, 4H), 3.98 (m, 12H), 3.57 (t, 28H), 3.29 (t, 4H), 2.29 (m, 4H), 1.98 (m, 6H), 1.75 (s, 24H), 1.54 (m, 4H), 1.36 (m, 8H). ^{13}C NMR (126 MHz, Chloroform-d) δ 180.26, 179.38, 170.43, 169.69, 156.83, 142.36, 127.85, 123.88, 122.36, 109.46, 86.68, 70.29, 64.63, 49.43, 43.67, 42.24, 40.78, 28.94, 27.2, 27.06, 26.76, 25.77, 16.17. HRMS (ES+) Calc. for $C_{92}H_{116}N_6O_{15}$ $[M+H]^+$ 1544.8499, found 1545.8536

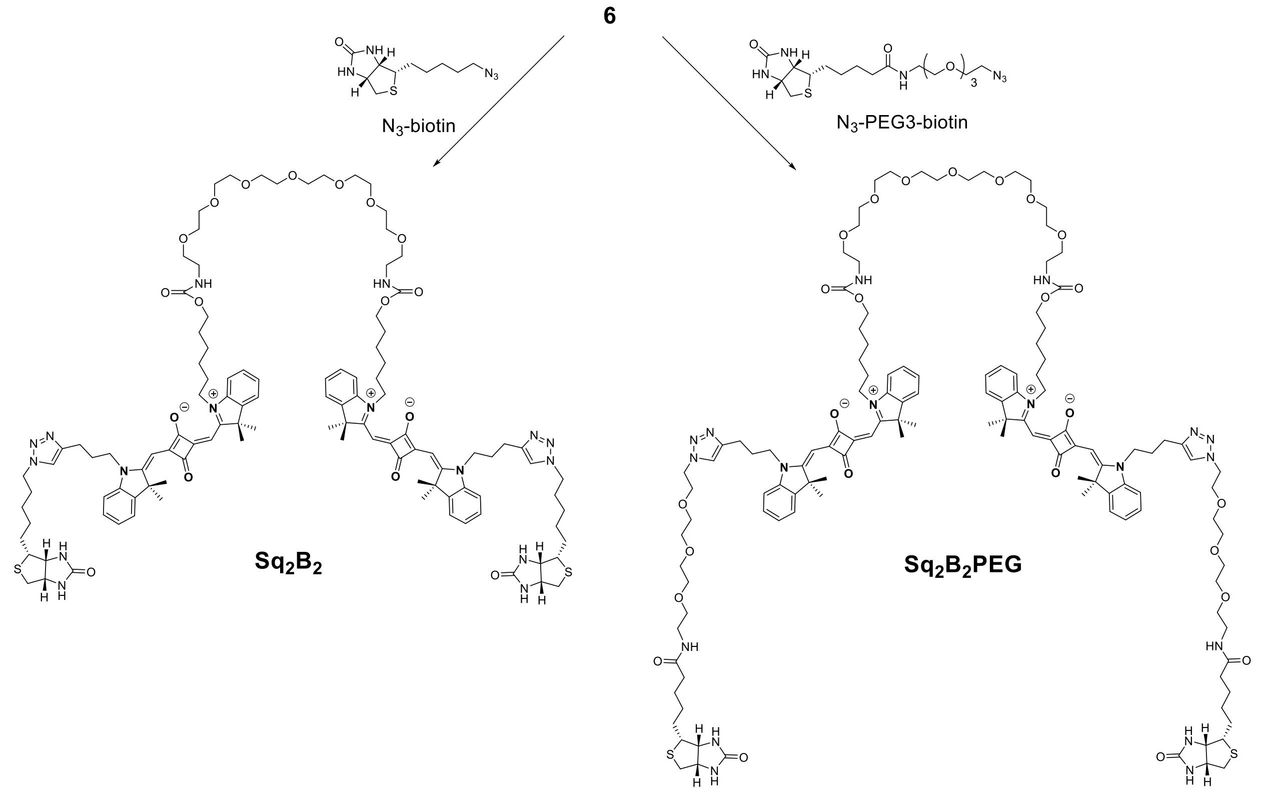

Scheme S3. Synthesis of Sq_2B_2 and Sq_2B_2PEG. $CuSO_4{\cdot}5H_2O$, sodium ascorbate, DMF/water (3/1), 60°C, 18h

General protocol for synthesis of Sq_2B_2 and Sq_2B_2PEG.
6 (1eq) and N_3-biotin (2.4 eq) or N_3-PEG3-biotin (2.4 eq) were dissolved in a mixture DMF/water (3/1). $CuSO_4{\cdot}5H_2O$ and sodium ascorbate were dissolved in an eppendorf with water and the solution was mixed until the mixture turn yellow. The content of the eppendorf was then added to the mixture and the

reaction was stirred at 60°C overnight. The solvent was then removed under vacuum, the crude product was dissolved in DCM, washed with 0.05 M EDTA solution, dried over MgSO4 and concentrated. The crude was purified by column chromatography in the gradient of eluent DCM/MeOH (9/1 to 7/3) to yield blue syrup (44% for Sq_2B_2, 70% for Sq_2B_2PEG)

Sq_2B_2 ^{1}H NMR (400 MHz, Chloroform-d) δ 7.54 (s, 2H), 7.28 (dd, J = 7.8, 6.6 Hz, 5H), 7.23 (dd, J = 7.7, 1.3 Hz, 3H), 7.07 (q, J = 7.1 Hz, 4H), 6.94 (dd, J = 16.5, 7.9 Hz, 4H), 5.98 (s, 2H), 5.87 (d, J = 11.3 Hz, 4H), 5.48 (d, J = 8.7 Hz, 3H), 4.45 – 4.35 (m, 2H), 4.32 (t, J = 7.2 Hz, 4H), 4.19 (dd, J = 8.5, 4.5 Hz, 2H), 4.04 – 3.84 (m, 14H), 3.55 (d, J = 7.4 Hz, 27H), 3.47 (t, J = 5.2 Hz, 4H), 3.41 (s, 1H), 3.28 (q, J = 5.6 Hz, 4H), 3.06 – 2.98 (m, 2H), 2.83 – 2.73 (m, 7H), 2.64 (d, J = 12.7 Hz, 2H), 2.22 – 2.04 (m, 14H), 1.84 (q, J = 7.3 Hz, 4H), 1.70 (d, J = 2.4 Hz, 26H), 1.60 – 1.48 (m, 7H), 1.35 (ddt, J = 24.5, 16.7, 7.2 Hz, 18H). ^{13}C NMR (101 MHz, Chloroform-d) δ 178.92, 170.15, 127.92, 127.85, 123.82, 122.32, 122.24, 121.55, 109.69, 109.46, 86.59, 70.51, 70.48, 70.23, 70.16, 64.62, 62.01, 60.14, 55.61, 50.12, 49.30, 43.62, 42.89, 40.76, 40.51, 29.93, 28.91, 28.47, 28.38, 27.10, 27.05, 26.99, 26.70, 26.41, 26.29, 25.73, 22.92. HRMS (ES+) Calc. for $C_{112}H_{150}N_{16}O_{14}S_2$ $[M+H]^+$ 2056.0806, found 2056.0832

Sq_2B_2PEG: ^{1}H NMR (500 MHz, Chloroform-d) δ 7.92 (s, 2H), 7.46 (t, 4H), 7.39-7.35 (q, 4H), 7.27-7.19 (m, 8H), 5.99 (d, 4H), 4,594 (t, 4H), 4.035 (t, 4H), 3.62-3.59 (m, 34H), 3.56-348 (m, 16H), 3.34 (p, 16H), 3.28 (t, 4H), 3.18 (p, 4H), 2.93-2.29 (m, 6H), 2.69 (d, 2H), 2.25-2.18 (m, 8H), 1.96 (s, 64H), 1.85-1.83 (m, 4H), 1.76 (d, 24H), 1.73-1.58 (m, 10H), 1.45-1.38 (p, 5H). ^{13}C NMR (126 MHz, Chloroform-d) δ 184.57, 180.42, 177.20, 176.56, 176.11, 172.51, 172.00, 166.12, 159.34, 143.72, 143.42, 143.26, 129.42, 129.41, 125.65, 125.50, 124.44, 123.51, 111.63, 111.46, 87.36, 87.22, 71.64, 71.56, 71.52, 71.42, 71.37, 71.28, 70.69, 70.52, 65.87, 63.47, 61.73, 57.11, 51.46, 50.74, 50.62, 44.79, 44.12, 41.73, 41.19, 40.46, 36.85, 30.88, 30.23, 29.90, 29.84, 29.63, 28.31, 27.84, 27.73, 27.57, 27.47, 26.96, 23.80, 23.71, 23.67. HRMS (ES+) Calc. for $C_{128}H_{180}N_{18}O_{25}S_2$ $[M+H]^+$ 2433.2808, found 2433.2624

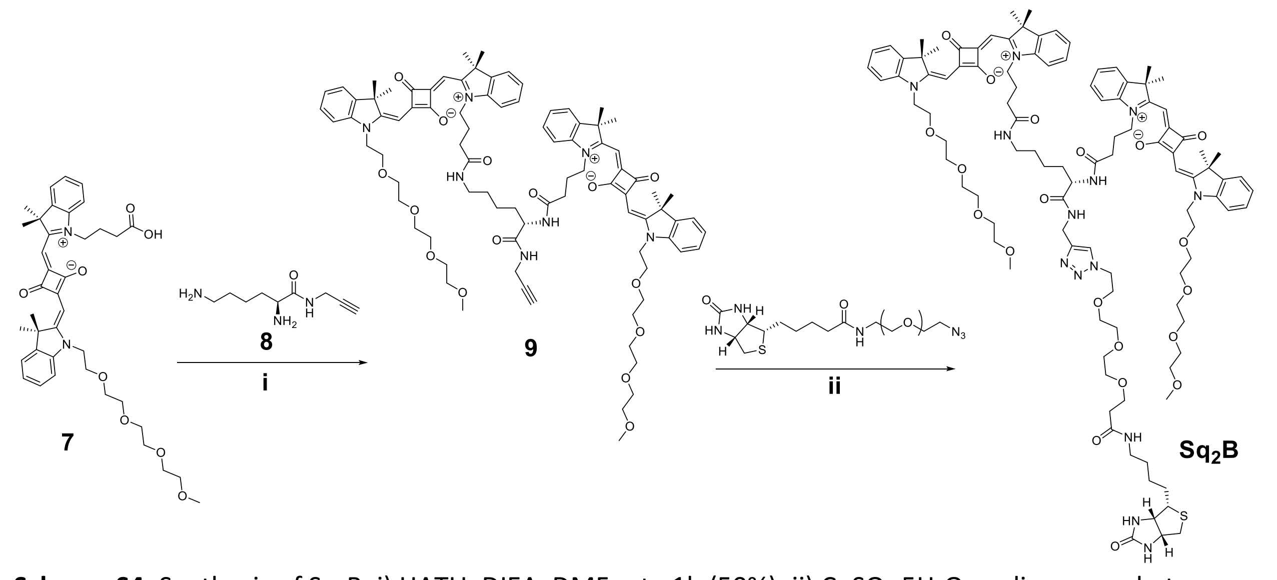

Scheme S4. Synthesis of Sq_2B. i) HATU, DIEA, DMF, r.t., 1h (50%); ii) $CuSO_4 \cdot 5H_2O$, sodium ascorbate, DMF/water (3/1), 60°C, 18h (60%).

Compound **7** and **8** were synthesized according to a published protocols.[4,5]

Synthesis of compound 9. To a solution of **7** (40 mg, 0.06 mmol, 2 eq) and **8** (12 mg, 0.03 mmol, 1 eq) in DMF (3 mL) was added HATU (54 mg, 0.14 mmol, 1.2 eq) followed by DIEA (62 μL, 0.36 mmol, 12 eq). After 1 h the solvents were evaporated and the crude was first purified by column chromatography on silica gel (DCM/MeOH: 8/2). Yield 50%.

^{1}H NMR (500 MHz, Chloroform-d) δ 10.96 (s, 1H), 7.96 (s, 1H), 7.53 (s, 2H), 7.32 – 7.26 (m, 0H), 7.28 – 7.20 (m, 5H), 7.21 (d, J = 1.6 Hz, 2H), 7.22 – 7.16 (m, 1H), 7.09 (d, J = 7.9 Hz, 1H), 7.10 – 7.00 (m, 7H), 6.93 (d, J = 14.6 Hz, 1H), 5.93 (d, J = 8.2 Hz, 2H), 5.86 (s, 2H), 4.48 (q, J = 7.4 Hz, 1H), 4.17 (s, 5H), 4.04 (s, 3H), 3.92 (ddd, J = 8.8, 5.4, 2.5 Hz, 2H), 3.77 (td, J = 5.9, 1.7 Hz, 4H), 3.62 (p, J = 6.7 Hz, 2H), 3.55 – 3.41 (m, 22H), 3.28 (s, 5H), 3.21 (dq, J = 13.9, 6.8 Hz, 2H), 3.04 (q, J = 7.4 Hz, 2H), 2.42 (t, J = 6.7 Hz, 2H), 2.37 (t, J = 6.9 Hz, 2H), 2.11 – 1.96 (m, 2H), 1.79 (t, J = 7.2 Hz, 1H), 1.66 (d, J = 12.7 Hz, 25H), 1.51 (dq, J = 21.9, 7.7, 7.3 Hz, 2H), 1.42 – 1.27 (m, 17H), 1.20 (d, J = 15.5 Hz, 6H), 0.80 (q, J = 10.5, 8.6 Hz, 1H). ^{13}C NMR (126 MHz, Chloroform-d) δ 172.32, 142.94, 142.06, 128.09, 127.72, 124.12, 123.81, 123.73, 122.25, 122.02, 110.24, 110.09, 109.85, 79.70, 71.84, 71.82, 71.17, 71.05, 70.54, 70.39, 70.37, 67.65, 59.01, 53.58, 49.49, 49.43, 49.23, 43.91, 43.17, 42.95, 41.96, 38.76, 32.83, 32.42, 30.31, 29.69, 29.03, 28.61, 27.12, 26.95, 22.80, 22.61, 22.52, 18.57, 17.45, 11.84. Calc. for $C_{87}H_{109}N_7O_{15}$ $[M]^+$ 1492.7982, found 1491.7989.

Synthesis of **Sq$_2$B** and **SqB** was done following general protocol for synthesis of **Sq$_2$B$_2$** and **Sq$_2$B$_2$PEG.**
Sq$_2$B: ^{1}H NMR (400 MHz, Chloroform-d) δ 10.04 – 9.99 (m, 15H), 8.04 (d, J = 6.8 Hz, 1H), 7.94 (s, 1H), 7.72 (d, J = 7.3 Hz, 1H), 7.52 (d, J = 6.0 Hz, 1H), 7.40 (s, 1H), 7.32 (td, J = 11.7, 9.6, 5.2 Hz, 9H), 7.18 (qd, J = 8.9, 7.7, 3.9 Hz, 9H), 6.52 (s, 3H), 6.07 (s, 1H), 6.02 (s, 1H), 5.94 (d, J = 14.5 Hz, 2H), 4.62 – 4.55 (m, 1H), 4.59 – 4.47 (m, 4H), 4.39 (dd, J = 8.0, 4.8 Hz, 2H), 4.25 (d, J = 5.6 Hz, 4H), 4.07 (t, J = 8.1 Hz, 4H), 3.95 (s, 1H), 3.84 (d, J = 5.4 Hz, 6H), 3.53 (ddt, J = 10.0, 6.6, 4.1 Hz, 35H), 3.46 – 3.38 (m, 2H), 3.34 (s, 6H), 3.18 (dq, J = 18.7, 6.7 Hz, 3H), 2.90 (dd, J = 13.2, 4.8 Hz, 1H), 2.76 (d, J = 13.0 Hz, 1H), 2.52 (t, J = 7.3 Hz, 4H), 2.24 (t, J = 7.5 Hz, 2H), 2.08 (q, J = 8.7, 7.8 Hz, 5H), 1.79 (s, 1H), 1.70 (s, 17H), 1.62 (t, J = 7.4 Hz, 1H), 1.57 – 1.49 (m, 1H), 1.46 – 1.38 (m, 1H), 1.37 (s, 3H). ^{13}C NMR (126 MHz, Chloroform-d) δ 175.53, 172.86, 142.02, 141.86, 141.58, 128.04, 125.35, 125.19, 124.90, 122.20, 122.09, 114.23, 111.19, 111.11, 110.90, 110.65, 71.79, 71.00, 70.98, 70.51, 70.49, 70.41, 70.37, 70.24, 70.00, 69.27, 68.80, 67.75, 62.49, 60.92, 58.91, 55.41, 51.03, 49.90, 49.85, 49.67, 49.65, 44.43, 43.38, 40.30, 39.67, 39.43, 35.16, 34.04, 32.44, 32.10, 31.20, 28.23, 27.92, 27.80, 26.54, 26.47, 26.43, 25.40, 23.35, 22.71. HRMS (ES+) Calc. for $C_{105}H_{141}N_{13}O_{20}S$ $[M+H+Na]^+$ 1960.0137, found 1960.0110
SqB: ^{1}H NMR (400 MHz, Methanol-d4) δ 8.16 – 8.08 (m, 2H), 7.78 (s, 1H), 7.35 – 7.27 (m, 4H), 7.25 – 7.15 (m, 3H), 7.08 (ddt, J = 8.8, 7.8, 1.4 Hz, 3H), 5.92 (s, 1H), 5.83 (s, 1H), 4.50 – 4.44 (m, 2H), 4.37 – 4.33 (m, 1H), 4.28 – 4.22 (m, 4H), 4.16 (dd, J = 7.9, 4.4 Hz, 1H), 4.08 (td, J = 6.8, 3.0 Hz, 2H), 3.84 – 3.75 (m, 4H), 3.61 – 3.53 (m, 2H), 3.50 – 3.43 (m, 6H), 3.36 (t, J = 5.4 Hz, 2H), 3.21 (dt, J = 3.3, 1.6 Hz, 6H), 2.82 – 2.72 (m, 3H), 2.08 (dt, J = 14.7, 7.3 Hz, 4H), 1.63 (d, J = 4.2 Hz, 12H), 1.49 (dd, J = 14.7, 7.2 Hz, 2H), 1.30 (q, J = 7.5 Hz, 2H). ^{13}C NMR (101 MHz, Methanol-d4) δ 183.05, 179.81 – 173.30 (m), 171.04 (d, J = 87.9 Hz), 164.63, 155.68, 152.55, 146.22, 146.14 – 141.27 (m), 127.76 (d, J = 23.7 Hz), 124.87, 123.97 (d, J = 5.0 Hz), 122.88, 110.38 (d, J = 84.5 Hz), 85.77, 73.13 – 63.93 (m), 61.08 (d, J = 175.8 Hz), 55.59, 50.76 – 48.90 (m), 43.22 (d, J = 118.9 Hz), 39.30 (d, J = 72.7 Hz), 35.33, 28.23 (d, J = 27.2 Hz), 26.31, 25.73 (d, J = 60.7 Hz), 22.29. HRMS (ES+) Calc. for $C_{57}H_{78}N_8O_{11}S$ $[M+Na]^+$ 1105.5511, found 1105.5420.

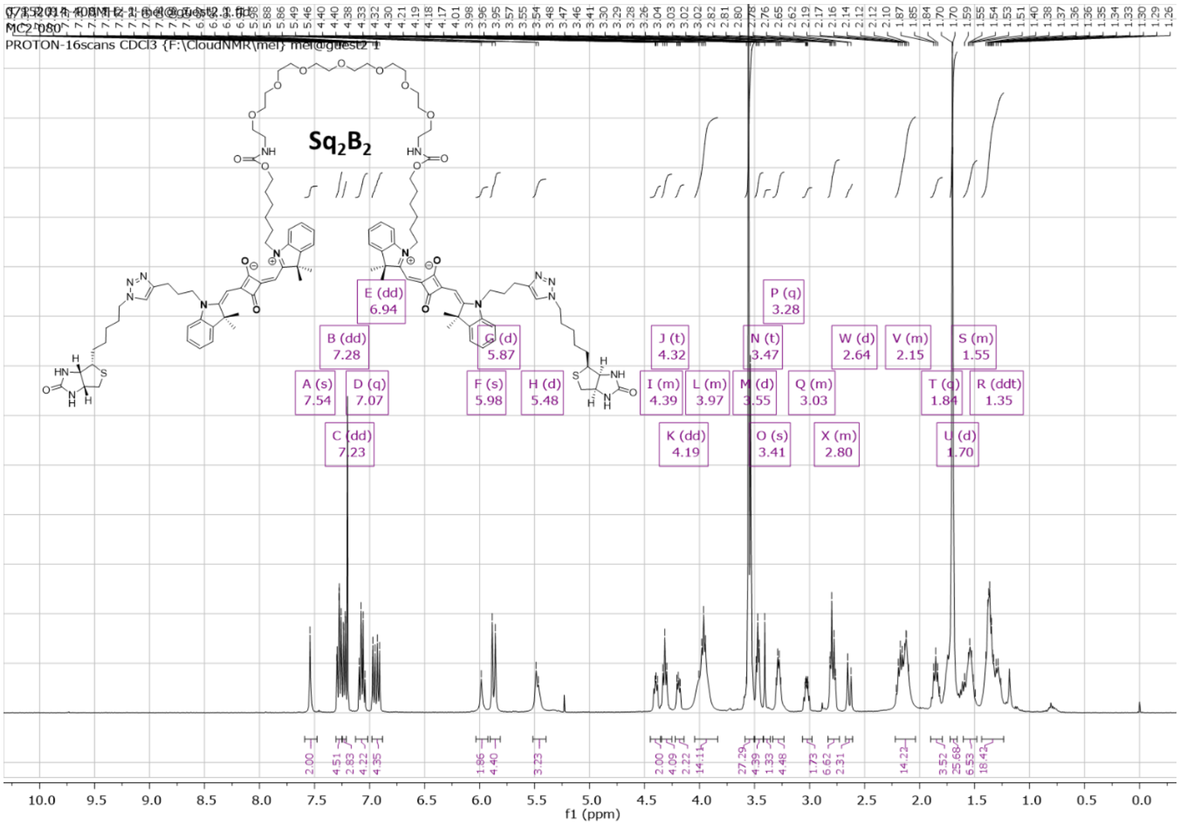

^{1}H NMR spectrum of Sq_2B_2

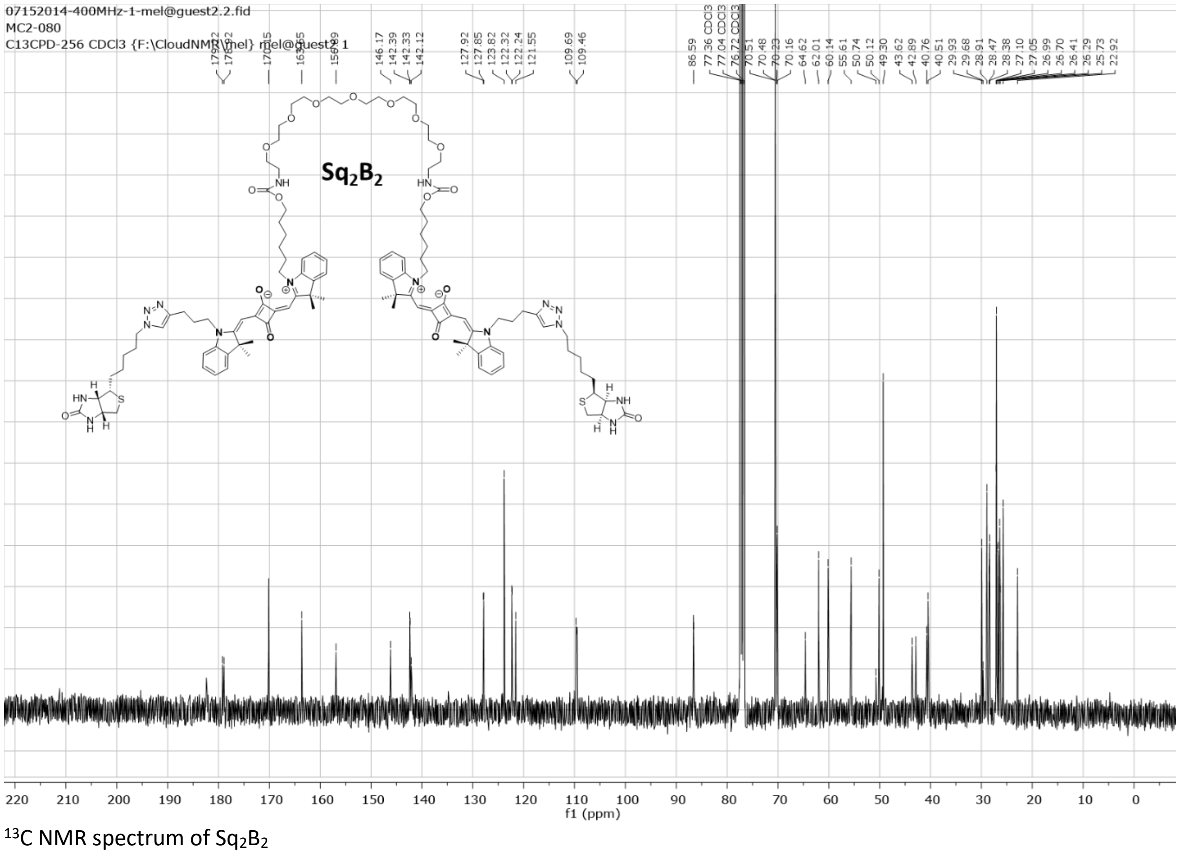

^{13}C NMR spectrum of Sq_2B_2

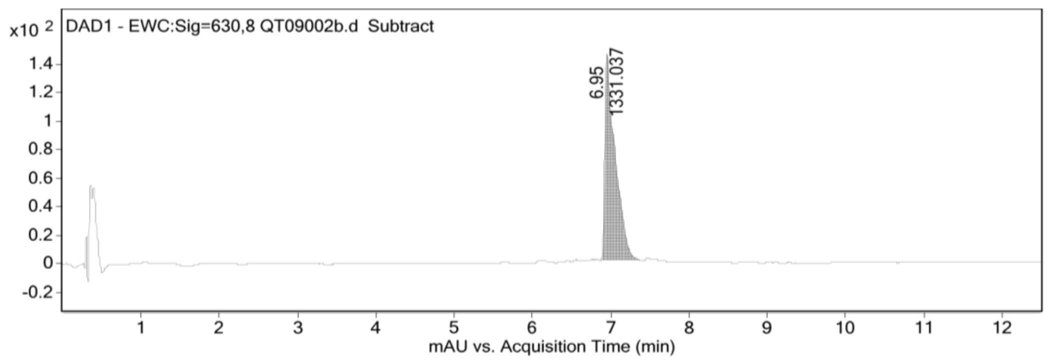

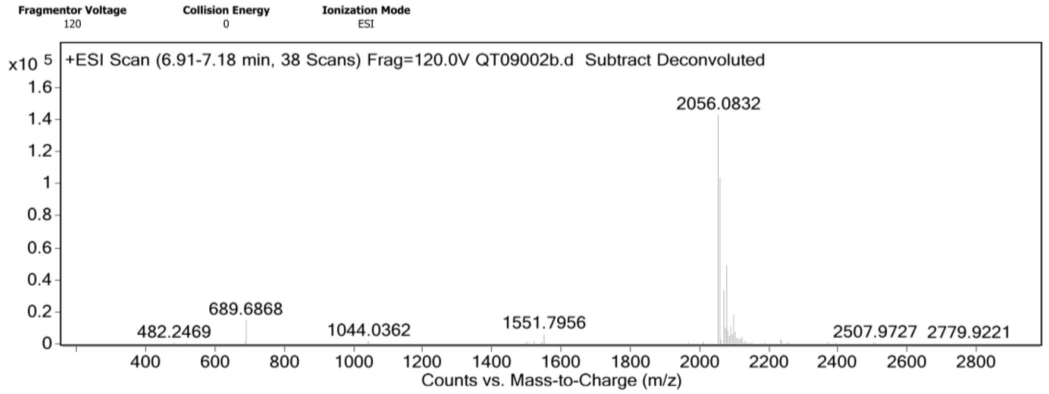

HP-LC chromatogram and HR-MS spectrum of Sq_2B_2

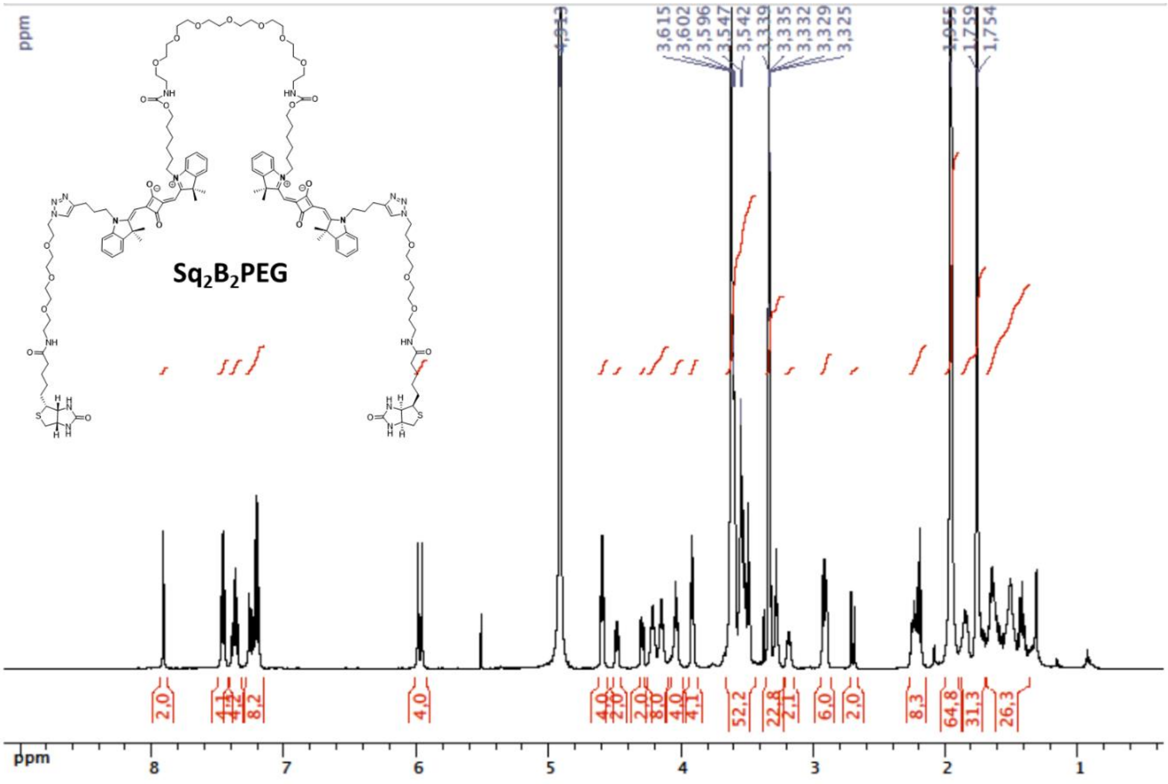

^{1}H NMR spectrum of Sq_2B_2PEG

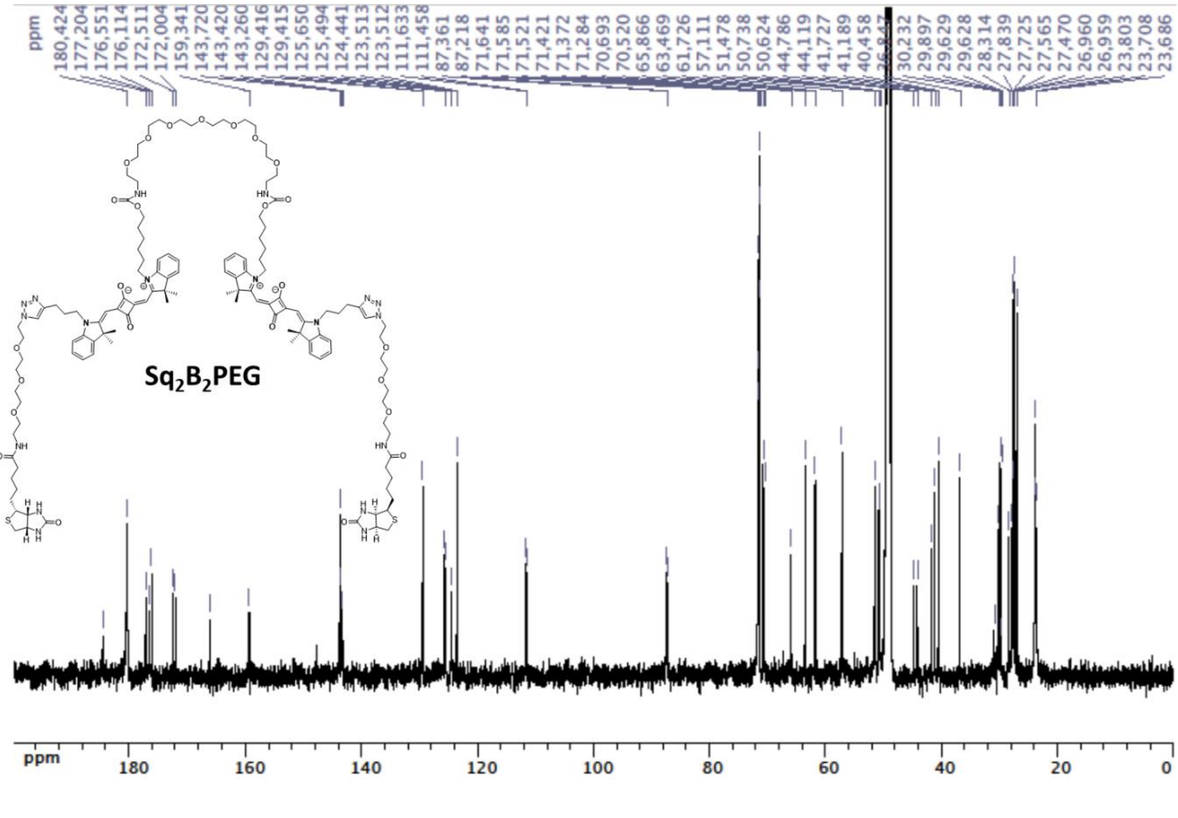

^{13}C NMR spectrum of Sq_2B_2PEG

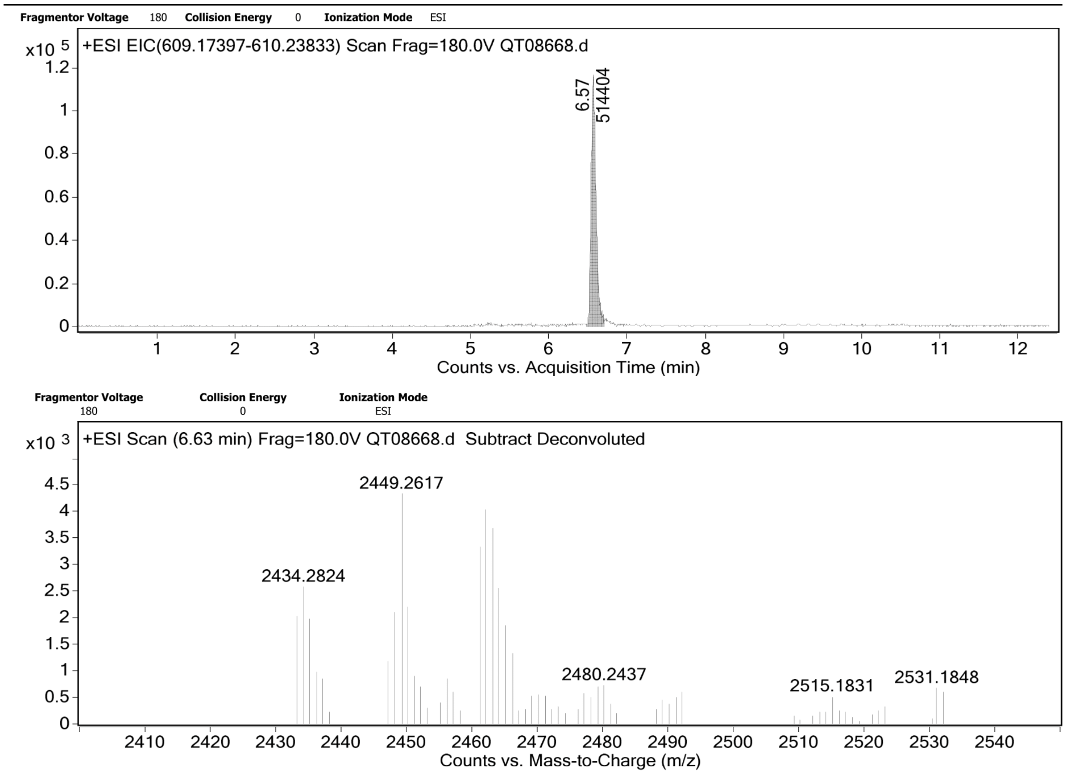

HP-LC chromatogram and HR-MS spectrum of Sq_2B_2PEG

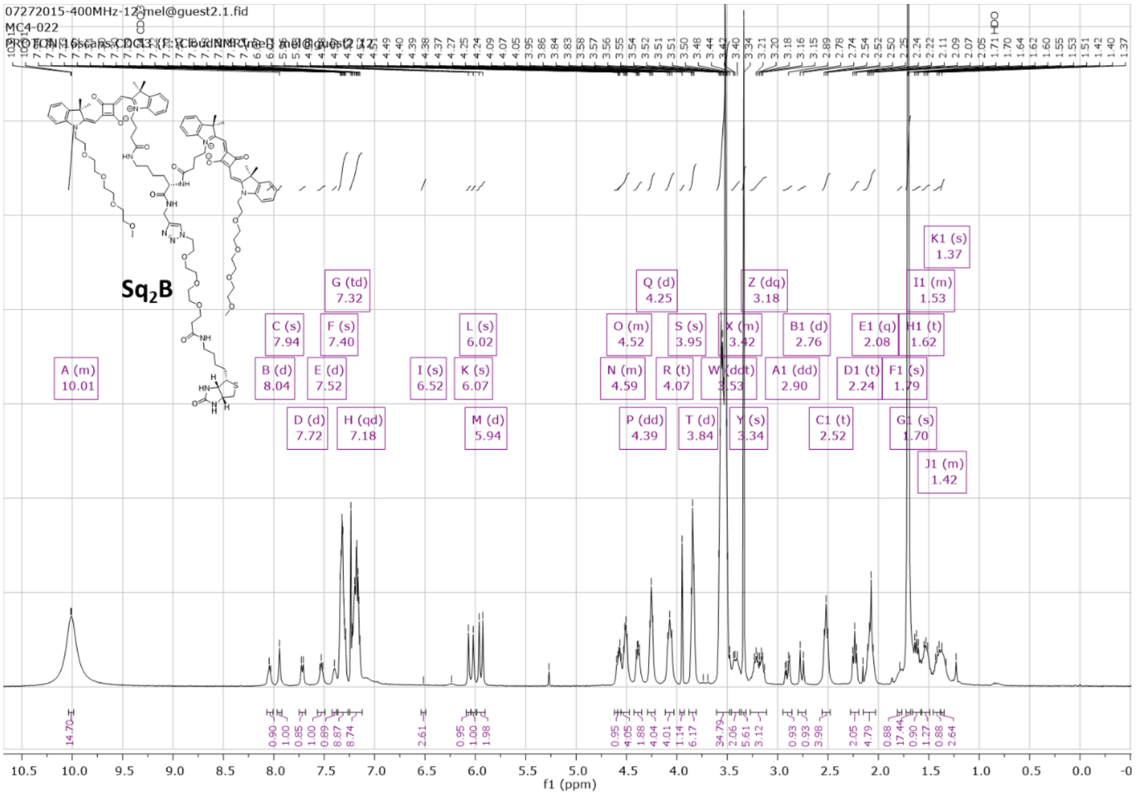

^{1}H NMR spectrum of Sq_2B

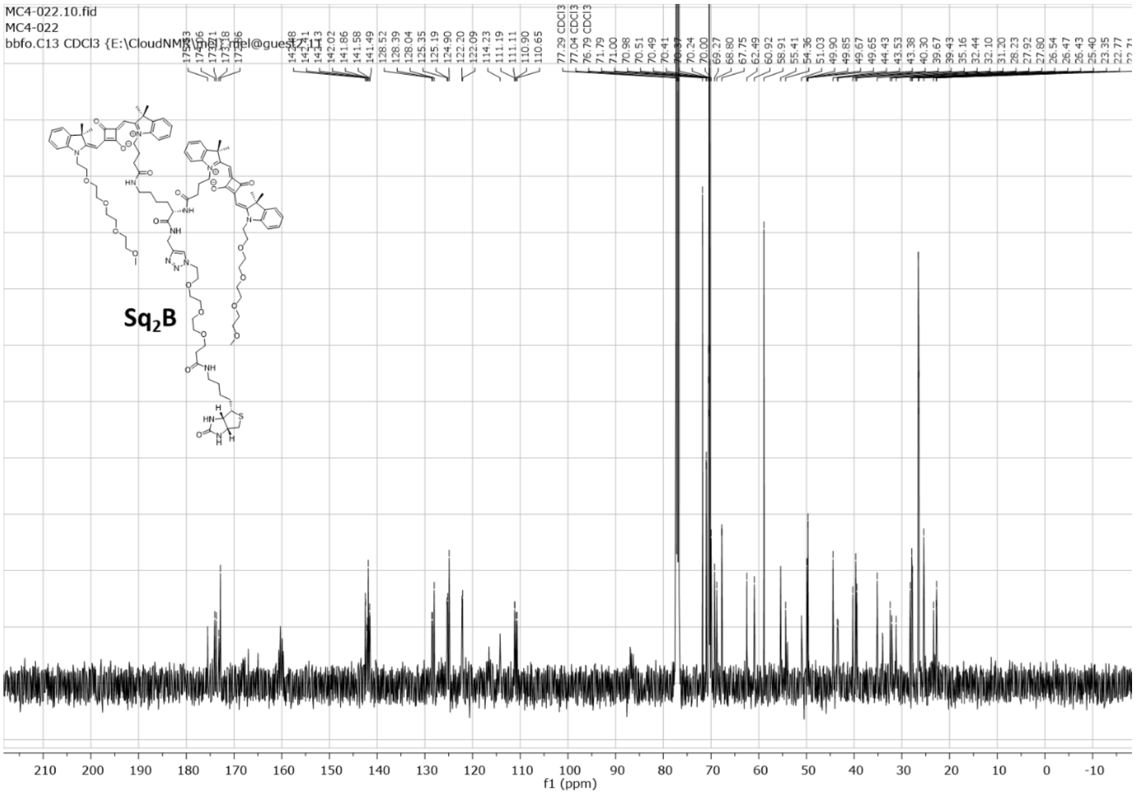

^{13}C NMR spectrum of Sq_2B

Fragmentor Voltage	Collision Energy	Ionization Mode
380	0	ESI

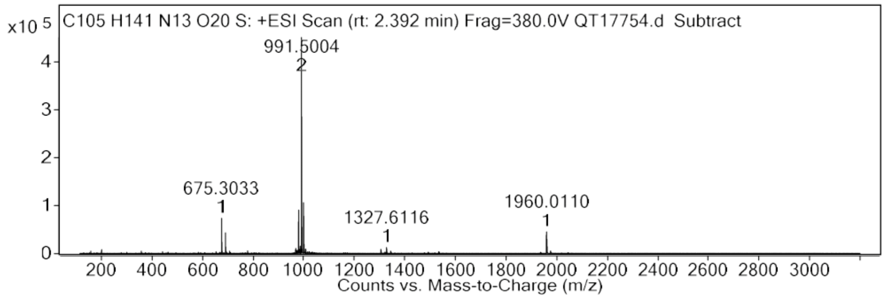

HR-MS spectrum of Sq_2B

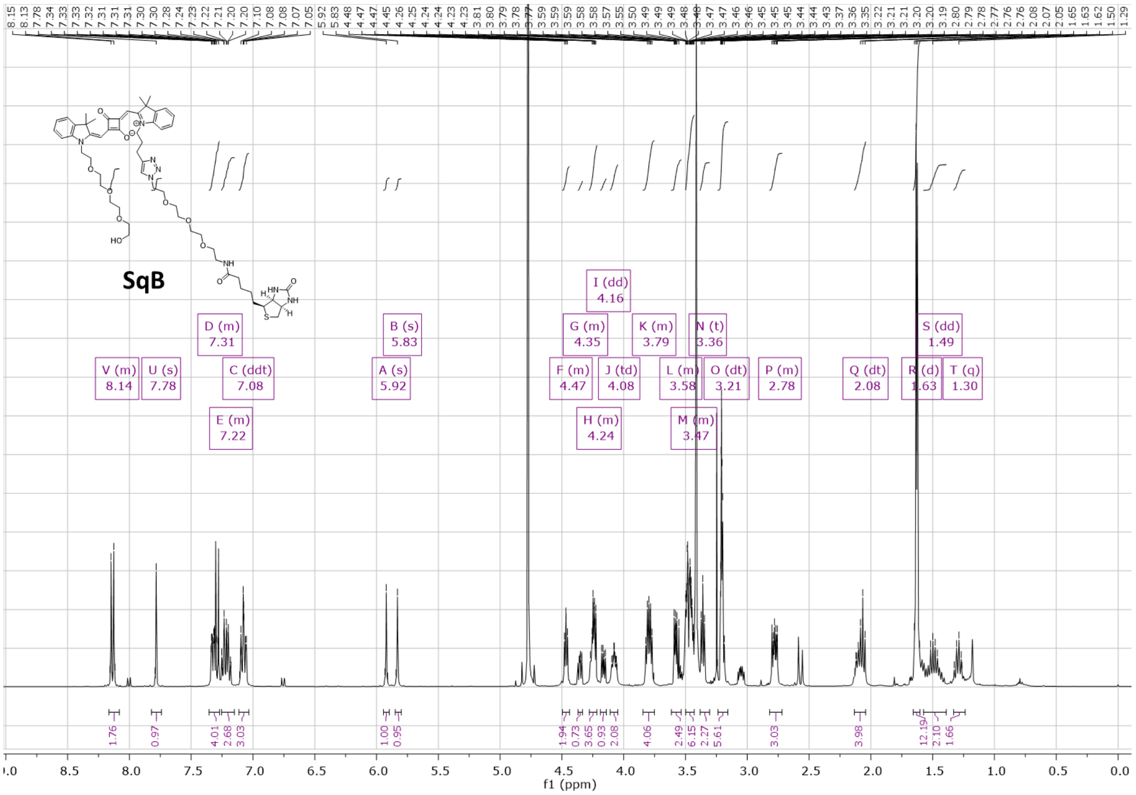

^{1}H NMR spectrum of SqB

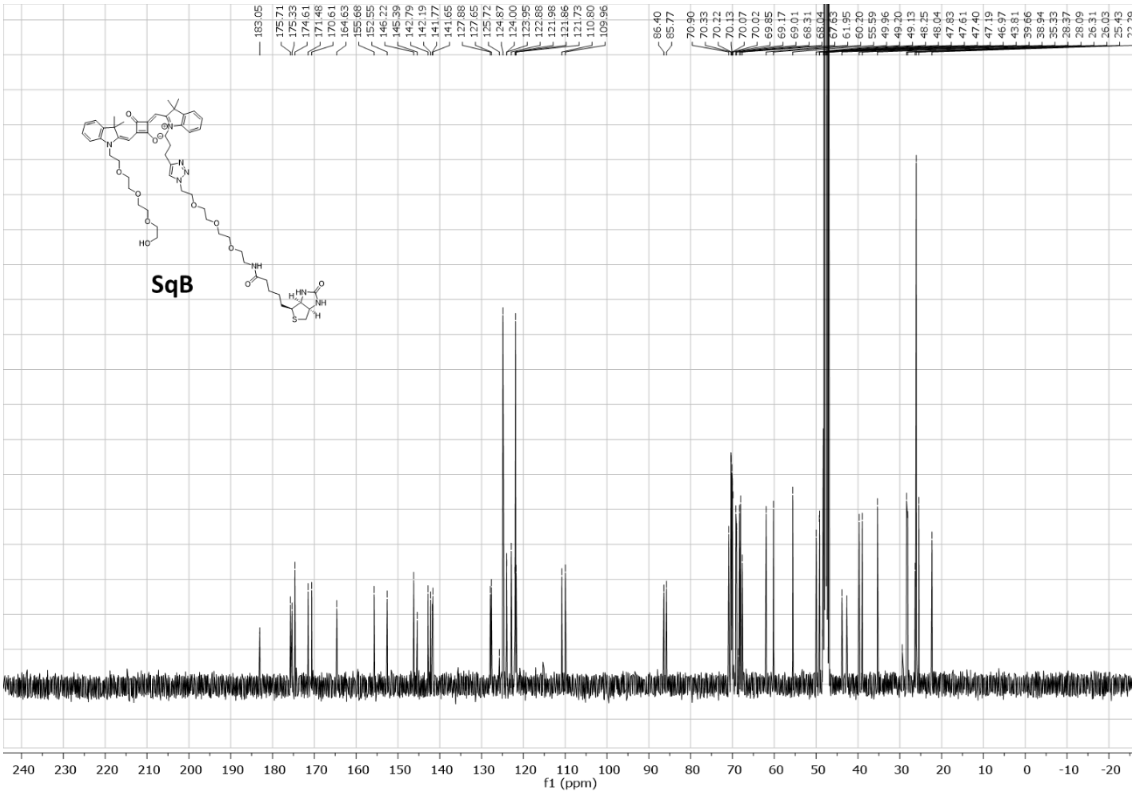

^{13}C NMR spectrum of SqB

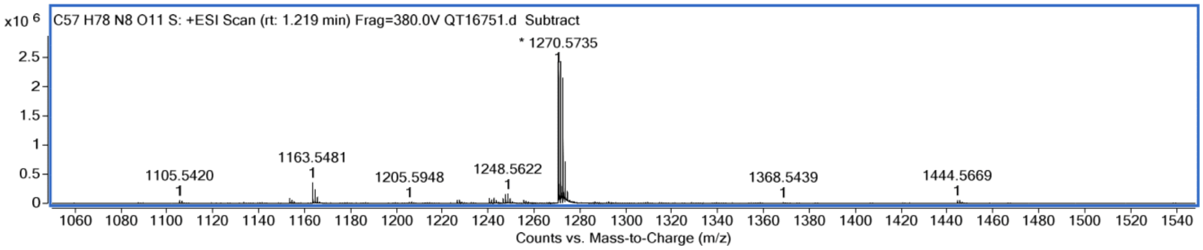

HR-MS spectrum of SqB

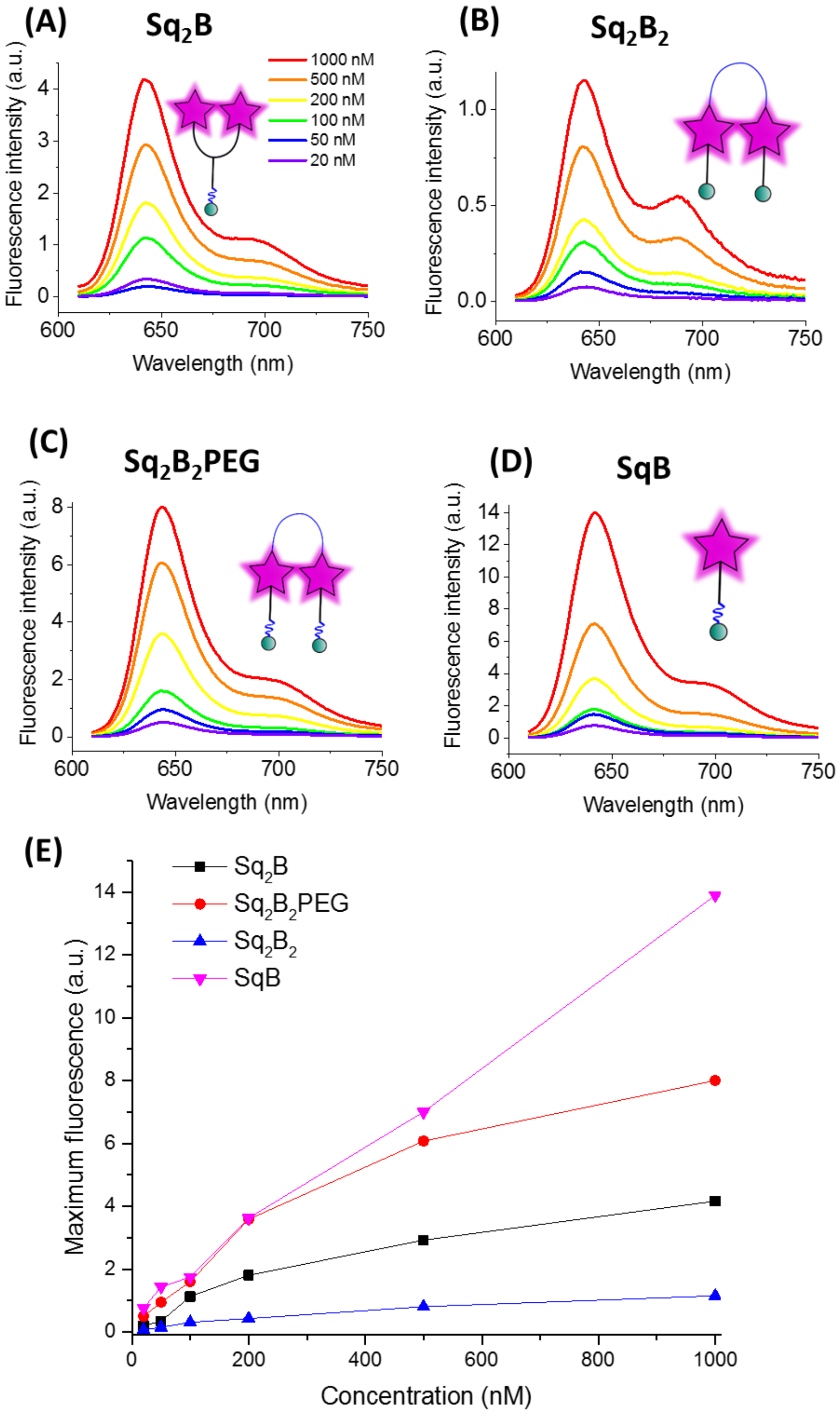

Fig. S1. Fluorescence spectra of Sq_2B (A), Sq_2B_2 (B), Sq_2B_2PEG (C) and SqB (D) at different concentrations in water. (E) Plot of fluorescence maxima from A-D of Sq2B, Sq2B2, Sq2B2PEG and SqB.

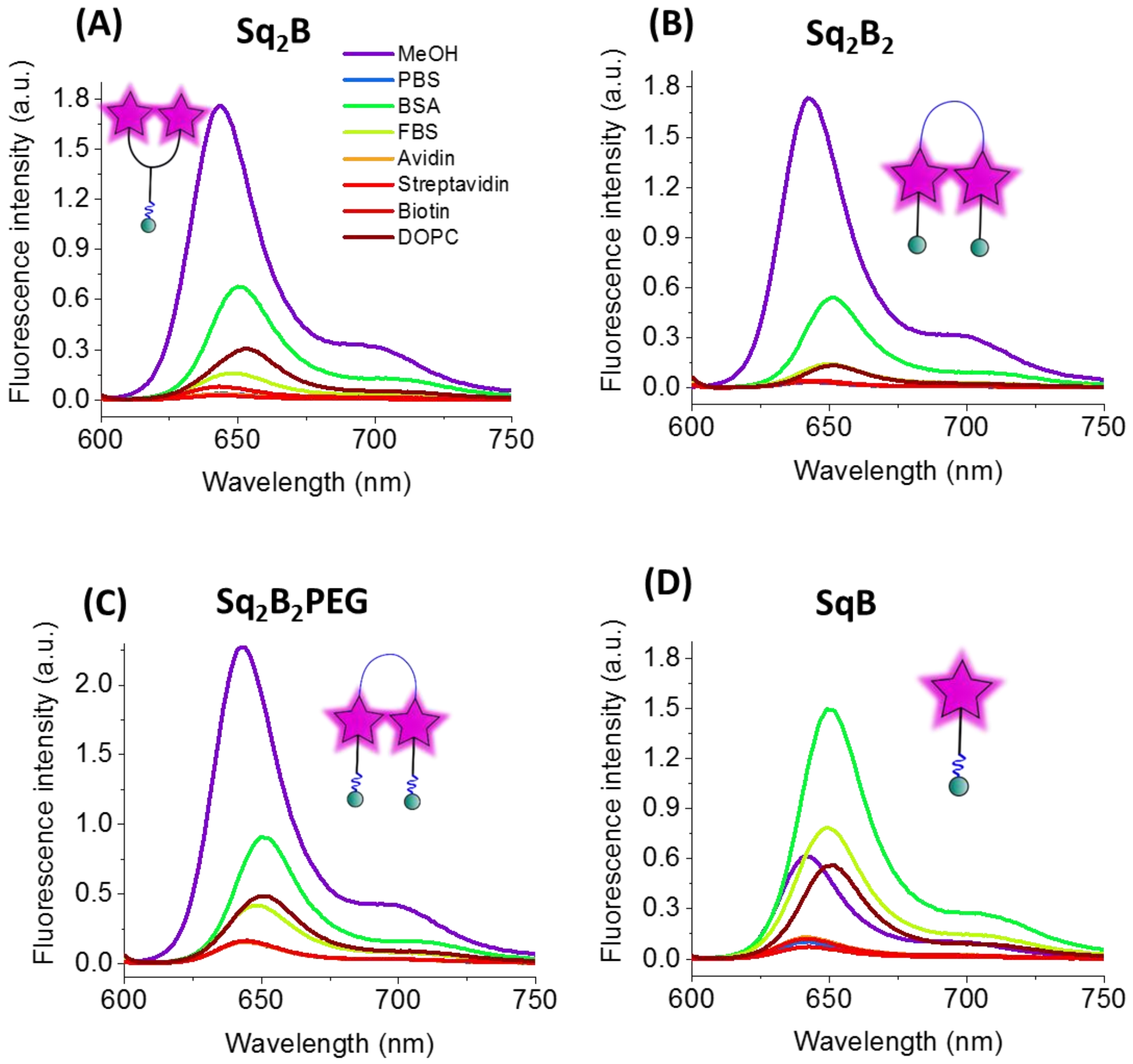

Fig. S2. Fluorescence spectra of 0.2 µM of Sq_2B (A), Sq_2B_2 (B), Sq_2B_2PEG (C) and SqB (D) in MeOH, PBS, in the presence of BSA (0.1 mg/mL), FBS (0.1 mg/mL), avidin (100 nM), streptavidin (100 nM), biotin (100 µM) or DOPC (20 µM).

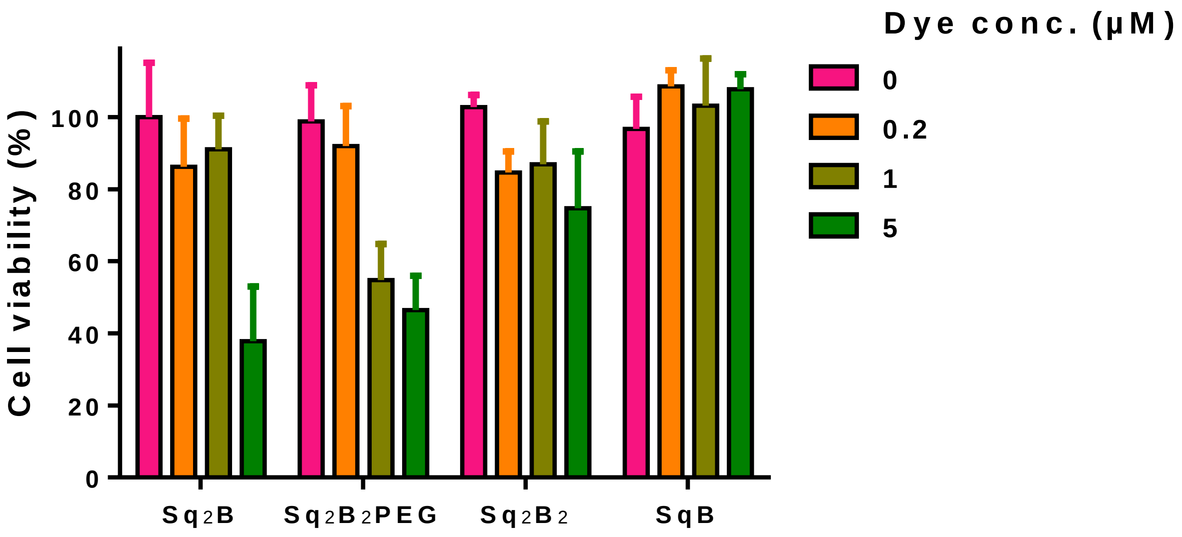

Fig. S3. Cytotoxicity of the probes after 24h incubation with KB cells determined by the MTT assay.

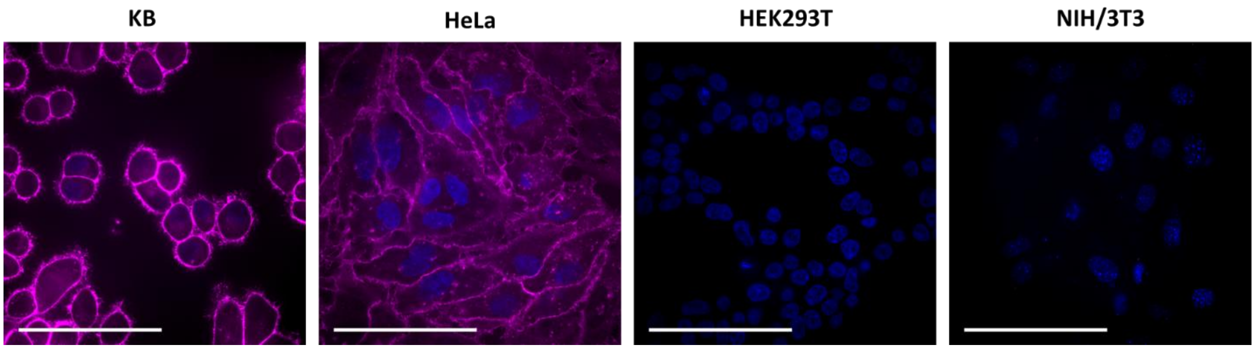

Fig. S4. Live cell fluorescence imaging of Sq_2B (0.2 µM) in cancer HeLa, KB cells and non-cancer HEK293T and NIH/3T3 cells. In all cases, the probe was incubated for 5 min at rt. Nuclei were labelled with Hoescht (5 µg/mL). Scale bar, 100 µm.

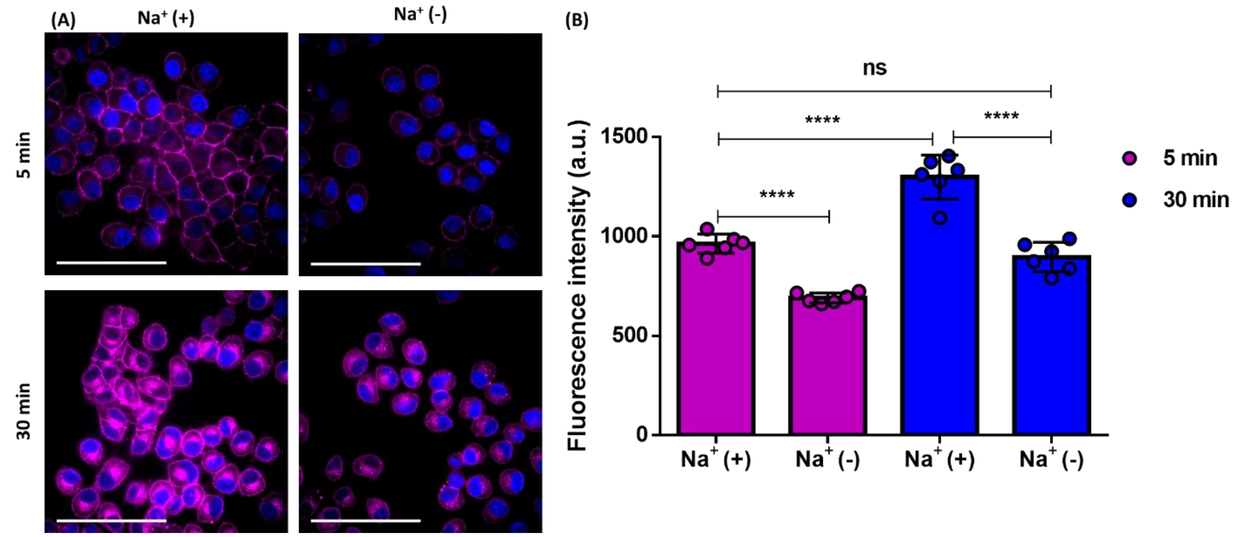

Fig. S5. Effect of Na^+-depletion in the buffer on internalization of Sq_2B. (A) Live-cell fluorescence imaging in KB cells of Sq_2B (0.2 µM) incubated for 5 or 30 min in Na^+-HBSS or Na^+-depleted HBSS buffer (Na^+ was replaced with choline) respectively. In all cases, the temperature was 37°C. Nuclei were labelled with Hoescht (5 µg/mL). Scale bar, 100 µm. (B) Analysis of fluorescence intensities from images (A). Bars represent mean value ± S.D. (n=6). Statistical significance based on unpaired t-test analysis: ****$p<0.0001$, ns - not significant.

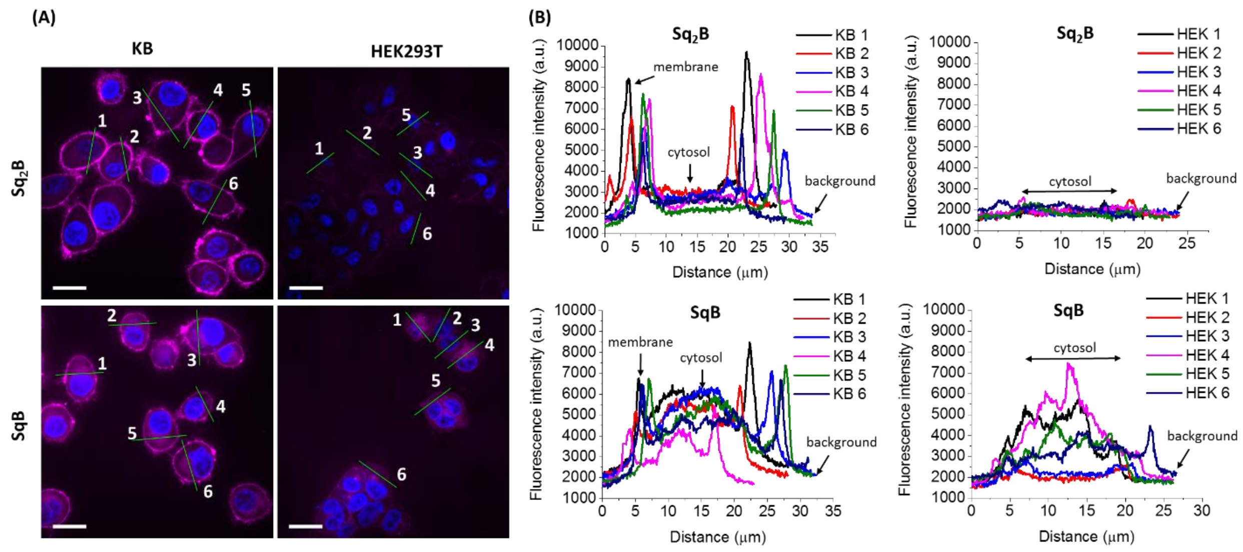

Fig. S6. Comparison of Sq_2B and SqB in cell imaging experiments. (A) Live-cell fluorescence images. Top panels: Sq_2B (0.2 µM) in BR-positive (KB) and BR-negative (HEK293T) cells. Bottom panels: SqB (0.2 µM) in BR-positive (KB) and BR-negative (HEK293T) cells. In all cases, the probe was incubated for 5 min at rt. Nuclei were labelled with Hoescht (5 µg/mL). Scale bar, 20 µm. (B) Fluorescence intensity profiles along ROIs (green lines) across individual cells (n=6) from images A.
